# Meta-Analysis of qPCR for Bovine Respiratory Disease Based on MIQE Guidelines

**DOI:** 10.3389/fmolb.2022.902401

**Published:** 2022-07-18

**Authors:** Rebecca J. Barnewall, Ian B. Marsh, Jane C. Quinn

**Affiliations:** ^1^ School of Agricultural, Environmental and Veterinary Science, Charles Sturt University, Wagga Wagga, NSW, Australia; ^2^ Gulbali Institute, Wagga Wagga, NSW, Australia; ^3^ NSW DPI, Elizabeth Macarthur Agricultural Institute, Menangle, NSW, Australia

**Keywords:** bovine respiratory disease, BRDC, cattle, molecular diagnostics, PCR, MIQE, qPCR, quantification

## Abstract

Qualitative and quantitative PCR-based tests are widely used in both diagnostics and research to assess the prevalence of disease-causing pathogens in veterinary medicine. The efficacy of these tests, usually measured in terms of sensitivity and specificity, is critical in confirming or excluding a clinical diagnosis. We undertook a meta-analysis to assess the inherent value of published PCR diagnostic approaches used to confirm and quantify bacteria and viruses associated with bovine respiratory disease (BRD) in cattle. This review followed the Preferred Reporting Items for Systematic Reviews and Meta-Analyses (PRISMA) guidelines. A thorough search of nine electronic databases (Web of Science, EBSCOhost, Cambridge journals online, ProQuest, PubMed, Sage journals online, ScienceDirect, Wiley online library and MEDLINE) was undertaken to find studies that had reported on the use of PCR and/or qPCR for the detection and/or quantification of BRD associated organisms. All studies meeting the inclusion criteria for reporting quantitative PCR for identification of BRD associated microorganisms were included in the analysis. Studies were then assessed on the applications of the Minimum Information for Publication of Quantitative Real-Time PCR Experiment (MIQE) and PCR primer/probe sequences were extracted and tested for *in silico* specificity using a high level of stringency. Fourteen full-text articles were included in this study. Of these, 79% of the analysed articles did not report the application of the MIQE guidelines in their study. High stringency *in silico* testing of 144 previously published PCR primer/probe sequences found many to have questionable specificity. This review identified a high occurrence of primer/probe sequences with a variable *in silico* specificity such that this may have implications for the accuracy of reporting. Although this analysis was only applied to one specific disease state, identification of animals suspected to be suffering from bovine respiratory disease, there appears to be more broadly a need for veterinary diagnostic studies to adopt international best practice for reporting of quantitative PCR diagnostic data to be both accurate and comparable between studies and methodologies.

## 1 Introduction

Polymerase chain reaction (PCR) is increasingly being adopted as the method of choice for disease detection and diagnosis in both human and veterinary medicine. In recent times PCR has been crucial in rapidly detecting and thus reducing the spread of COVID-19 (Udugama et al., 2020; Kevadiya et al., 2021; Mardian et al., 2021). For the most part, PCR is used qualitatively, presence or absence. However, disease diagnosis can be further explored through the application of quantitative PCR, which can give information about the load of the infectious agent (Bhullar et al., 2014; Abdullah et al., 2018; Fabri et al., 2021). This technique can be particularly useful where normal flora is associated with the etiology of disease, for example, bovine respiratory disease (BRD) (Pansri et al., 2020; Klompmaker et al., 2021). Recently in human medicine, qPCR has been used to fast track diagnosis and evaluate treatment of Leishmaniosis (Wu et al., 2020) as well as differentiate disease from colonization of Pneumocystis pneumonia (Tan et al., 2021). Increasingly, PCR and qPCR are being adopted to increase sensitivity and specificity of disease diagnosis, beyond traditional gold-standard diagnostic techniques.

A high level of diagnostic sensitivity and specificity is essential when diagnosing disease to reduce the proportion of false-positive/negative results. In 2009, the MIQE guidelines were established (Bustin et al., 2009) to provide a guideline against which qPCR could be benchmarked. Though the recommendations are frequently referred to in the PCR community, primer and probe sequences are often reported that demonstrate limited or questionable stringent *in-silico* testing. Moreover, sensitivity and specificity are often not reported, significantly impeding the reader’s ability to interpret the prevalence and concentration of pathogens reported from a diagnostic sample.

Bovine respiratory disease is a complex disease where a definitive diagnosis is commonly conferred from non-specific clinical signs as well as situational characteristics. The bacterial species commonly associated with BRD which are also normal flora of the bovine upper respiratory tract include; *Mannheimia haemolytica*, *Pasteurella multocida*, *Histophilus somni* (Mahony and Horwood, 2007; Amat, 2019) and *Trueperella pyogenes* (Anholt et al., 2017; Cockcroft et al., 2020). These organisms have the potential to become opportunistic pathogens under certain conditions when viral infection and/or stress from necessary management practices cause coincident physiological compromise (Lubbers and Turnidge, 2015). For this reason, the detection of BRD-associated bacteria and viruses using PCR-based approaches is often complicated due to their commensal nature (Holman et al., 2015; Zeineldin et al., 2017). This complex mix of bacterial and viral pathogens often makes determining a definitive PCR diagnosis of active or subclinical disease exceptionally challenging. Therefore, a combined qualitative and quantitative approach is required for optimal disease incidence and prevalence reporting (Pansri et al., 2020).

To address the question of the rigor applied to published reports using PCR-based approaches to test for the bacteria and viruses associated with respiratory disease in cattle, a critical review was undertaken to determine the reliability and efficacy of published PCR-based tests due to the growing use and reliance of these techniques in disease diagnosis. *In silico* testing was applied to primer and probe sequences to determine their specificity. Our findings suggest that specificity and sensitivity of currently published PCR-based tests for bovine respiratory disease are highly variable and that more stringent application of the MIQE guidelines would increase the reliability and reproducibility or reported assays.

## 2 Methods

### Search Terms and Screening Procedure

This meta-analyses was undertaken using the Preferred Reporting Items for Systematic Reviews and Meta-Analyses (PRISMA) guidelines (Page et al., 2021) using NVivo (QSR International Pty Ltd, 2012).

The PICOS (Population, Intervention, Comparison, Outcome, Study design) (Tacconelli, 2009) theoretical framework was used to define the research question. The objective of the review was to determine the frequency of published reports showing alignment to MIQE guidelines that reported on the use of PCR based tests for BRD bacterial and viral associated organisms. The *in silico* specificity of primer/probe sequences was then analysed to determine rates of potential false-positive results reported in those articles. *Population:* Although “population” is not a well-aligned term in this instance, reports were included where molecular tests were reported in the peer-reviewed literature that used PCR-based approaches for identification and/or quantification of BRD associated viral and bacterial organisms in cattle. Articles that identified other species or applications were excluded. *Interventions:* Any PCR test reported to identify and/or quantify BRD associated viral and bacterial organisms in cattle. *Outcome*: In this context, the outcome was a positive PCR-based test for BRD associated organisms that had been reported in the peer-reviewed literature. *Study design*: Clinical trials and research methodologies were included in the analysis providing specific information was provided on primer/probe sequence data.

### Information Sources

Nine databases (Web of Science, EBSCOhost, Cambridge journals online, ProQuest, PubMed, Sage journals online, ScienceDirect, Wiley online library and MEDLINE) were interrogated in May and November 2021 using the search terms: 2010 to 2021, peer-reviewed; “bovine respiratory disease” OR BRD OR “shipping fever” AND cattle OR bovine AND “polymerase chain reaction” OR PCR OR qPCR OR RT-PCR OR RT-qPCR. All search results were imported into Endnote and duplicates were removed. Pneumonia was not included as a search term as pneumonia can be caused by other ailments not just BRD and this would have resulted in over 25,000 search results, lots of which didn’t report on the implication of pneumonia in BRD and therefore were not relevant to this study.

### Report Characteristics

During the initial screening, only title and abstracts were examined in Endnote. Inclusion criteria were the study reported on the use of PCR for diagnosis or detection of bovine respiratory disease associated pathogens; was published between 2010–2021; was not a review article and reported on the bovine species only.

### Study Selection

**FIGURE 1:**
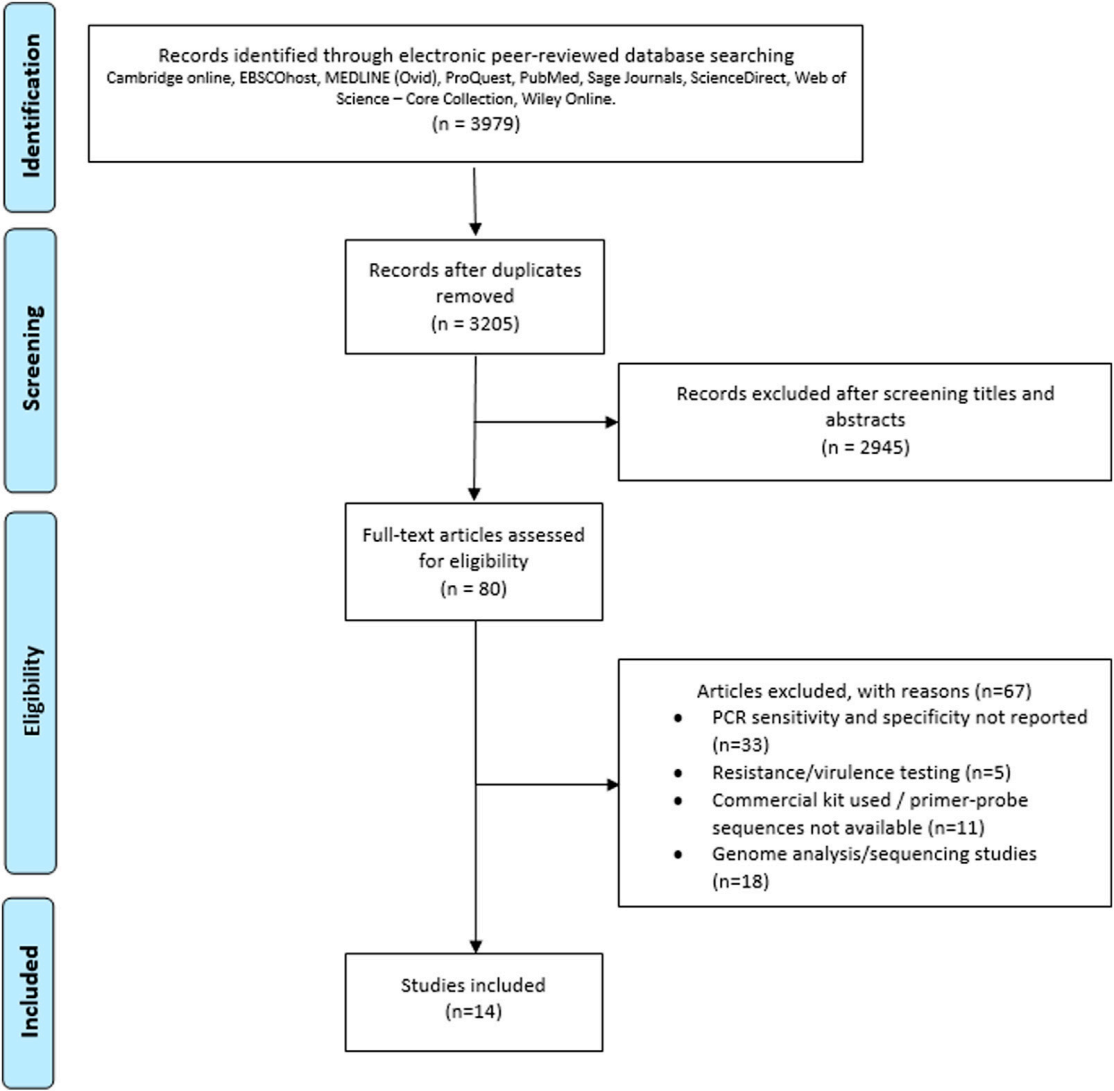
PRISMA flow chart of search, inclusion and exclusion screening and accepted studies of the review of the use of PCR for diagnosis and/or detection of pathogens associated with bovine respiratory disease (Page et al., 2021).

The second round of screening was undertaken to identify articles directly related to the study protocol, this was undertaken by two independent assessors examining the title, abstract, methods and results of each article. Definitions of commonly used PCR terms in this study were based on those reported in the MIQE guidelines (Table 1). Exclusion criteria included: studies not related to bovine respiratory disease; studies not reporting sensitivity and specificity testing of molecular tests; studies not reporting PCR probe/primer sequence data; studies reporting resistance/virulence testing only but not pathogen detection; and studies reporting genome/sequencing analysis alone e.g. for identification of resistance genes but not specifically microorganism identification. Studies were included if sufficient data was presented on primer/probe sequences to undertake *in silico* analysis, and/or analytical and diagnostic sensitivity and specificity were reported to have been undertaken. Case studies or case series reports were excluded due to the high risk of potential bias. Only studies reported in English, and where a full text was available, were included. Full-text articles remaining after the two-phase screening and eligibility determination (n = 14) were included in this review. Reference lists from those articles were also scanned for additional relevant publications. Information on the application of the MIQE guidelines was extracted and tabulated for each article, along with reported primer/probe sequence data. Search terms and the number of articles retained in the study are shown in Figure 1 (Page et al., 2021).

**TABLE 1:** Definitions used in this study, based on the MIQE Guidelines (Bustin et al., 2009).

Term	Definition
Analytical Sensitivity	Minimum number of copies in a sample that can be detected
Analytical Specificity	Ability to only detect the true target of the test
Clinical (diagnostic) Sensitivity	Percentage of true positives samples in a population detected by a test
Clinical (diagnostic) Specificity	Percentage of true negative samples in a population identified by a test as negative
Accuracy	The difference in the measured and actual concentration
Repeatability	The ability to get the same result with the same test at the same time
Reproducibility	The ability to get the same result with the same test over multiple runs overtime

### *In silico* Specificity

Specificity of individual primer and probe sequences were tested *in silico,* between May - November 2021*,* by screening against sequences lodged in the NCBI BLASTn Nucleotide collection (nr/nt) database (Madden, 2003). *In silico* analysis of published primer/probe sequences was undertaken to determine the number of target-specific and non-target specific sequences identified for each primer/probe sequence by alignment with known sequences within the database. A threshold of 5,000 targets was used as the upper limit, a number significantly higher than the default setting in BLASTn, to ensure that all putative sequences were identified and to avoid limiting hits to the most prevalent sequences lodged in the database. Reporting bias for the reduction in sequence identities sourced by BLASTn analysis was achieved by application of query cover and identity threshold above 95% sequence specificity.

Primer-Blast (Ye et al., 2012) was then used for simultaneous assessment of primer sets. For the broadest coverage the nr database was chosen and no organism was specified. The maximum target amplicon size used was the pre-set value of 4,000. All degenerate bases in primer sequences (W, R, Y) were changed to “N”, a requirement of the Primer-Blast software. The number of Blast hits analysed, which is determined by the software for each search, was recorded for each primer pair.

### Application of the MIQE Guidelines

The application and reporting of how the MIQE guidelines were applied was analysed based on the criteria in Table 2 (Nour et al., 2014). Each reported methodology was considered in detail against the reporting criteria. If all criteria were met (Table 2) the article was considered to have applied the MIQE guidelines.

**TABLE 2:** Quality and application with MIQE guidelines. Modified from Nour et al. (2014).

Items	Analysed Parameters	Method of Analysis
Assay details	Primer (probe) sequences/assay ID	“yes” if primer (and probe) sequences are provided
	PCR efficiency	“yes” if this is an assessment of amplification efficiency
	Assay specificity	“yes” if there is mention of *in silico* homology search (BLAST, ePCR, BiSearch, or alike)
Reverse transcription	Input amount of RNA in RT reaction	“yes” if input amount of RNA in RT reaction is mentioned
	RT enzyme or RT kit	“yes” if there is any mention of reverse transcriptase used or specific kit, along with minimal instructions (can be according to manufacturer)
	Priming method	“yes” if the type of primers is mentioned (random primers, oligo-dT, blend, gene-specific primers,…)
PCR	PCR conditions	“yes” if PCR conditions are listed or referred to an older publication
	Taq polymerase or PCR kit	“yes” if there is any mention of Taq polymerase used or specific kit, along with minimal instructions (can be according to manufacturer)
	Final primer concentration	“yes” if final primer concentration in the reaction is mentioned (or can be deduced)
	Input amount template in PCR reaction	“yes” if input amount of template is mentioned
qPCR validation	Evidence of optimization	‘yes’ if protocol optimization outlined
	Evidence of LOD (analytical sensitivity)	“yes” if the mention of serial dilution of known target

### Statistical Analysis

Fisher’s exact test (Jones et al., 2012; Yoon et al., 2021) was applied to determine if there was a statistically significant difference (i.e., if *p* < 0.05) in target-specific and non-target search results from BLASTn analysis, using the free professional statistical software R (R Development Core Team, 2020).

## 3 Results

A total of 3,979 records were imported into EndNote (EndNote Team, 2013) from the following nine online databases; Cambridge online (41), EBSCOhost (1,359), MEDLINE (131), ProQuest (1,029), PubMed (131), Sage Journals (60), ScienceDirect (349), Web of Science–Core Collection (423) and Wiley Online (456). After the removal of 954 duplicates, 3,025 records were identified using the initial search terms. During screening of titles and abstracts, 2,945 records were excluded, leaving 80 records to be assessed for eligibility. The following exclusion criteria were applied, and 67 articles were excluded; not reporting PCR sensitivity and specificity (n = 33), reporting on resistance/virulence testing (n = 5), using a commercial kit/primer-probe sequences were not available (n = 11) or reporting on genome analysis/sequencing (n = 18). Articles reporting resistance/virulence testing were excluded as detection of genes associated with resistance and virulence does not infer pathogen presence. No discrimination was made based on origin of clinical sample, cohort size or PCR reaction type (singleplex or multiplex). Fourteen full-text records were exported into NVivo for analysis (Figure 1). All studies included primer/probe sequence data or clearly referenced cited studies. All articles included both analytical sensitivity and specificity and only one study included diagnostic sensitivity and specificity (Andres-Lasheras et al., 2020); ten studies reported on PCR optimization (Horwood and Mahony, 2011; Thonur et al., 2012; Nefedchenko et al., 2016; Fan et al., 2017; Zhang et al., 2017; Loy et al., 2018; Thanthrige-Don et al., 2018; Liu et al., 2019; Andres-Lasheras et al., 2020; Xie et al., 2021) and three studies reported the use of quantitative PCR methods; qPCR (Andres-Lasheras et al., 2020), Multiplex qRT-PCR (Goto et al., 2020) and RT-qPCR (Xie et al., 2021). Quantitative values were not reported in any of the studies included in this analysis (Table 3).

**TABLE 3:** Reporting of optimization, analytical and diagnostic sensitivity and specificity, PCR method, quantification and MIQE guideline application (Table 2). Se, sensitivity; Sp, specificity.

PCR Method Reported	Optimization	Analytical		Diagnostic		Quantification	MIQE Guidelines Applied	References
		Se	Sp	Se	Sp			
qPCR	**✓**	**✓**	**✓**	**✓**	**✓**	**✗**	**✓**	Andres-Lasheras et al. (2020)
Multiplex qRT-PCR	**✗**	**✓**	**✓**	**✗**	**✗**	**✗**	**✗**	Goto et al. (2020)
Multiplex real-time RT-PCR	**✓**	**✓**	**✓**	**✗**	**✗**	**✗**	**✗**	Horwood and Mahony, (2011)
Taqman real-time PCR	**✗**	**✓**	**✓**	**✗**	**✗**	**✗**	**✗**	Kishimoto et al. (2017)
NanoPCR	**✓**	**✓**	**✓**	**✗**	**✗**	**✗**	**✗**	Liu et al. (2019)
Multiplex real-time PCR	**✓**	**✓**	**✓**	**✗**	**✗**	**✗**	**✓**	Loy et al. (2018)
PCR & RT-PCR (both multiplex)	**✓**	**✓**	**✓**	**✗**	**✗**	**✗**	**✗**	Thanthrige-Don et al. (2018)
Multiplex RT-PCR	**✓**	**✓**	**✓**	**✗**	**✗**	**✗**	**✓**	Thonur et al. (2012)
RT-qPCR	**✓**	**✓**	**✓**	**✗**	**✗**	**✗**	**✗**	Xie et al. (2021)
Multiplex PCR	**✓**	**✓**	**✓**	**✗**	**✗**	**✗**	**✗**	Zhang et al. (2017)
RT-PCR	**✗**	**✓**	**✓**	**✗**	**✗**	**✗**	**✗**	Socha and Rola, (2011)
GeXP-multiplex PCR	**✓**	**✓**	**✓**	**✗**	**✗**	**✗**	**✗**	Fan et al. (2017)
Multiplex PCR	**✓**	**✓**	**✓**	**✓**		**✗**	**✗**	Nefedchenko et al. (2016)
PCR	**✗**	**✓**	**✓**	**✗**	**✗**	**✗**	**✗**	Szacawa et al. (2015)

### Specificity of Published Primer/Probe Sequences for Microorganisms Associated With Bovine Respiratory Disease

*In silico* analysis was performed on all individual published primer/probes sequences (BLASTn), as well as for sequence target analysis of specific primer sets (Primer-Blast) to establish the frequency of target-specific and non-target results. BLASTn (Madden, 2003) search results with query coverage and identity above 95% were identified as either target-specific or non-target organisms depending on if the search result was that of the intended target or not. Tabulated data is shown in Supplementary Table S1, S2. Primer-Blast (Ye et al., 2012) search results were identified as either target-specific or non-target specific. In the case of viruses, alternative strain types were also identified. Non-target organism (i.e. non-bovine) were also noted. The number of non-target sequence hits as well as product length range was compared (Supplementary Table S3, S4).

#### 3.1.1 Primer/Probe Sequences for Identification of Viruses Associated With Respiratory Disease in Cattle

Nine of the 14 studies included in this analysis reported on the use of PCR for the detection of viral pathogens associated with BRD (Horwood and Mahony, 2011; Socha and Rola, 2011; Thonur et al., 2012; Fan et al., 2017; Kishimoto et al., 2017; Thanthrige-Don et al., 2018; Liu et al., 2019; Goto et al., 2020; Xie et al., 2021). A total of 81 primer/probe sequences were extracted from the published studies (probes n = 18, forward primers n = 31, reverse primers n = 32) and analysed using BLASTn (Madden, 2003) and Primer-Blast (Ye et al., 2012). One study reported two assays targeting the M gene of Bovine Parainfluenza Virus (BPIV-3) (Thanthrige-Don et al., 2018) using the same forward primer but different reverse primers, therefore this forward primer was only considered once in our analysis (Table 4 and Table 5).

**TABLE 4:** Published PCR primer and probe sequences for detection of viral pathogens associated with bovine respiratory disease complex. F′ forward; P′ probe, R’ reverse sequences.

Pathogen	Target Gene	Sequence (5’—3′)	References
Bovine Adenovirus 3	Hexon	F′ ATT​ACC​AGC​GTC​AAC​CTC​TAC	Kishimoto et al. (2017)
		P′ TCC​ACT​TTG​GAA​GCT​ATG​CTC​CGC	
		R′ CCG​CCG​AGA​GAT​AGT​CAT​TAA​A	
Bovine Adenovirus 7	Hexon	F′ CRAGGGAATAYYTGTCTGAAAATC	Kishimoto et al. (2017)
		P′ TTCATCWCTGCCACWCAAAGCTTTTTT	
		R′ AAGGATCTCTAAATTTYTCTCCAAGA	
Bovine Coronavirus	N (nucleocapsid protein)	F′ GGA​CCC​AAG​TAG​CGA​TGA​G	(Kishimoto et al., 2017; Goto et al., 2020)
		P′ ATT​CCG​ACT​AGG​TTT​CCG​CCT​GG	
		R′ GAC​CTT​CCT​GAG​CCT​TCA​ATA	
	N	F′ GCC​GAT​CAG​TCC​GAC​CAA​TC	Thanthrige-Don et al. (2018)
		R′ AGA​ATG​TCA​GCC​GGG​GTA​T	
Bovine Herpes Virus 1	gE (glycoprotein E)	F′ CAA​TAA​CAG​CGT​AGA​CCT​GGT​C	(Kishimoto et al., 2017; Goto et al., 2020)
		P′ TGC​GGC​CTC​CGG​GCT​TTA​CGT​CT	
		R′ GCT​GTA​GTC​CCA​AGC​TTC​CAC	
	gC	F′ ATG​TTA​GCG​CTC​TGG​AAC​C	Horwood and Mahony, (2011)
		P′ ACG​GAC​GTG​CGC​GAA​AAG​A	
		R′ CTT​TAC​GGT​CGA​CGA​CTC​C	
	Glycoprotein B gene	F′ TGA​GGC​CTA​TGT​ATG​GGC​AGT​T	Thanthrige-Don et al. (2018)
		R′ GGA​CAC​AAC​AAA​CAA​TGC​GG	
	Glycoprotein B gene	F′ TGT​GGA​CCT​AAA​CCT​CAC​GGT	Thonur et al. (2012)
		P′ AGG​ACC​GCG​AGT​TCT​TGC​CGC	
		R′ GTA​GTC​GAG​CAG​ACC​CGT​GTC	
	gB	F′ GCG​TCA​TTT​ACA​AGG​AGA​ACA​TC	Fan et al. (2017)
		R′ ATCTCGCCCATGCCCAC	
Bovine Influenza D Virus	PB1	F′ CAG​CTG​CGA​TGT​CTG​TCA​TAA​G	(Kishimoto et al., 2017; Goto et al., 2020)
		P′ AAT​GGA​CTT​TCT​CCT​GGG​ACT​GCT	
		R′ ACA​AAT​TCG​CAG​GGC​CAT​TA	
Bovine Parainfluenza Virus 3	M (membrane protein)	F′ TGT​CTT​CCA​CTA​GAT​AGA​GGG​ATA​AAA​TT	(Horwood and Mahony, 2011; Kishimoto et al., 2017; Goto et al., 2020)
		P′ ACA​GCA​ATT​GGA​TCA​ATA​A	
		R′ GCA​ATG​ATA​ACA​ATG​CCA​TGG​A	
	M gene	F′ TGTCTTCCACTMGATAGAGGGATAAAATT	Thanthrige-Don et al. (2018)
		R′ CCTTTCTCATCTAAGATCTGGACMACC	
		R′ CCT​TTT​TCA​TCT​AGA​ATC​TGA​ACT​ACT​CC	
	Nucleoprotein gene	F′ GGT​AGG​AGC​ACC​TCC​ACG​ATT	Thonur et al. (2012)
		P′ AAG​ATC​TTG​TTC​ACA​CAT​TC	
		R′ GCT​CCA​AGG​CAT​GCT​GGA​TA	
	Nucleoprotein gene	F′ TGA​TTG​GAT​GTT​CGG​GAG​TGA	Thonur et al. (2012)
		P′ TAC​AAT​CGA​GGA​TCT​TGT​TCA	
		R′ AGA​ATC​CTT​TCC​TCA​ATC​CTG​ATA​TAC​T	
Bovine Respiratory Syncytial Virus	N (nucleocapsid protein)	F′ GCA​ATG​CTG​CAG​GAC​TAG​GTA​TAA​T	(Kishimoto et al., 2017; Goto et al., 2020)
		P′ ACC​AAG​ACT​TGT​ATG​ATG​CTG​CCA​AAG​CA	
		R′ ACA​CTG​TAA​TTG​ATG​ACC​CCA​TTC​T	
	N gene	F′ TAT​GCT​ATG​TCC​CGA​TTG​G	Liu et al. (2019)
		R′ ACT​GAT​TTG​GCT​AGT​ACA​CCC	
	G attachment glycoprotein	F′ ACACATCAATYCAAAGCACCACAC	Thanthrige-Don et al. (2018)
		R′ GCTRGTTCTGTGGTGGRTTGTTGTC	
	Nucleocapsid	F′ GGT​CAA​ACT​AAA​TGA​CAC​TTT​CAA​CAA​G	Thonur et al. (2012)
		P′ TAGTACAGGTGACAA + CA + T + TG	
		R′ AGC​ATA​CCA​CAC​AAC​TTA​TTG​AGA​TG	
	Glycoprotein F	F′ AAT​CAA​CAT​GCA​GTG​CAG​TTA​G	Socha and Rola, (2011)
		R′ TTTGGTCATTCGTFATAGGCAT	
	Glycoprotein G	F′ CAT​CAA​TCC​AAA​GCA​CCA​CAC​TGT​C	Socha and Rola, (2011)
		R′ GCT​AGT​TCT​GTG​GTG​GAT​TGT​TGT​C	
	N4	F′ GTTGCTGCTTTGGTTAT	Socha and Rola, (2011)
		R′ AGACTTGTATGATGCTGC	
	N protein	F′ GTC​AGC​TTA​ACA​TCA​GAA​GTT​CAA​G	Socha and Rola, (2011)
		R′ ACA​TAG​CAC​TAT​CAT​ACC​ACA​ATC​A	

**TABLE 5:** Continued Published PCR primer and probe sequences for detection of viral pathogens associated with bovine respiratory disease complex. F′ forward; P′ probe, R’ reverse sequences.

Pathogen	Target Gene	Sequence (5’—3′)	References
Bovine Rhinitis A Virus	3Dpol	F′ CAC​CTG​AAC​TAT​GGA​CTT​GG	Kishimoto et al. (2017)
		P′ GAC​GTG​GAC​TGG​CAC​CAG​TTT​GC	
		R′ CACGGCCTCAATCATCTG	
Bovine Rhinitis B Virus	3Dpol	F′ AAC​GCG​ATT​GTG​TCC​TAG​GG	Kishimoto et al. (2017)
		P′ CTG​TCC​TTT​GCA​CGG​CGT​GG	
		R′ GCC​ACT​GAG​GTT​AGC​TTC​TC	
	3D gene	F′ CGTGGCACACTTCAGGAG	Xie et al. (2021)
		P′ TRGCRGGTCTCGCTTTYCACAGT
		R′ GTGTACCCAYCTCARACGAAG
Bovine Viral Diarrhea Virus	5′UTR	F′ GRAGTCGTCARTGGTTCGAC	Goto et al. (2020)
		P′ TGCYAYGTGGACGAGGGCATGC	
		R′ TCA​ACT​CCA​TGT​GCC​ATG​TAC	
	5′UTR	F′ TGG​ATG​GCT​TAA​GCC​CTG​AGT​A	Horwood and Mahony, (2011)
		P′ AGTCGTCAGTGGTTCGA
		R′ CCTCGTCCACGTGGCATC
	5′UTR	F′ GGGNAGTCGTCARTGGTTCG	Kishimoto et al. (2017)
		P′ CCAYGTGGACGAGGGCAYGC
		R′ GTGCCATGTACAGCAGAGWTTTT
	5′UTR	F′ CAT​ACC​TTC​AGT​AGG​ACG​AGC	Thanthrige-Don et al. (2018)
		R′ ATG​TGC​CAT​GTA​CAG​CAG​AG
	5′UTR	F′ CATGCCCRYAGTAGGACTAGC	Thanthrige-Don et al. (2018)
		R′ ATG​TGC​CAT​GTA​CAG​CAG​AG
	5′UTR	F′ GTGAGTTCGTTGGATGGC	Fan et al. (2017)
		R′ TAT​GTT​TTG​TAT​AAG​AGT​TCA​TTT​G

##### 3.1.1.1 BLASTn Analysis

**FIGURE 2:**
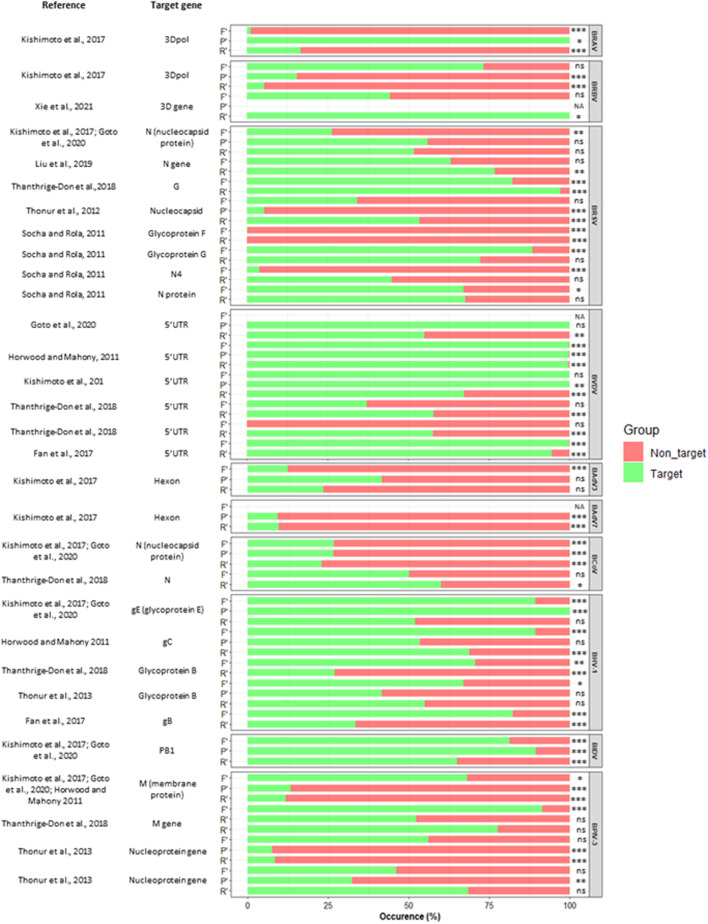
Occurrence of target and non-target sequence alignment for published primer and probe sequences targeting the respiratory disease-causing viral pathogens in cattle. Bovine coronavirus (BCoV), Bovine herpesvirus 1 (BHV-1), Bovine rhinitis A virus (BRAV), Bovine rhinitis B virus (BRBV), Bovine respiratory syncytial virus (BRSV) Bovine influenza D virus (BIDV) Bovine viral diarrhea virus (BVDV), Bovine parainfluenza virus 3 (BPIV-3), and Fisher’s exact test; *p* > 0.05 ns, *p* ≤ 0.05 *, *p* ≤ 0.01 **, *p* ≤ 0.001 ***.

Analysis of viral primer/probe sequences identified three sequences with 100% specificity for their intended target: probe for gE gene of BHV-1 (Kishimoto et al., 2017; Goto et al., 2020), probe for 3Dpol gene of BRAV (Kishimoto et al., 2017) and the reverse primer for 3D gene of BRBV (Xie et al., 2021). Three sequences returned no BLASTn search results at all for either target-specific nor non-target organisms: the forward primer targeting the Hexon gene of BAdV7 (Kishimoto et al., 2017) and probe targeting the 3D gene of BRBV (Xie et al., 2021), and the forward primer for 5′UTR gene of BVDV (Goto et al., 2020) (Figure 2). Of the 81 viral primer/probe sequences analysed, 59.3% returned a higher proportion of target-specific results than non-targets, of these, 66.7% had a significantly higher number of alignments for target-specific sequences than non-target. Thirty-one of the 81 sequences analysed (38.3%) had alignment results that identified a greater proportion of non-target than target specific hits (Figure 2 and Supplementary Table S1). Of these, 22 sequences (71.0%) had a significantly higher number of sequence hits for non-targets than target-specific (Figure 2). The primers and probe targeting the N (nucleocapsid protein) gene of BCoV (Kishimoto et al., 2017; Goto et al., 2020), and both primers targeting the Hexon gene of BAdV3 (Kishimoto et al., 2017) and both the Glycoprotein F gene N4 gene of BRSV (Socha and Rola, 2011), all returned significantly more results for non-targets than the target gene specified.

##### 3.1.1.2 Primer-Blast Analysis of Viral Primer/Probe Sequences

In general, viral primer/probe sequences showed good alignment to their intended target sequence, PrimerBlast analysis identified a number of published sequences that were 100% specific for their intended target, specifically BAdV3 Hexon (Kishimoto et al., 2017); BHV-1 gC (Horwood and Mahony, 2011); BIDV PB1 (Kishimoto et al., 2017; Goto et al., 2020); BRSV N (nucleocapsid protein) (Kishimoto et al., 2017; Goto et al., 2020), N gene (Liu et al., 2019) and G attachment glycoprotein (Thanthrige-Don et al., 2018); BRAV 3Dpol (Kishimoto et al., 2017); BRBV 3Dpol (Kishimoto et al., 2017) and 3D gene (Xie et al., 2021); BVDV 5′UTR (Horwood and Mahony, 2011) (Supplementary Table S3). Conversely, three primer sets returned no Primer-Blast hits for their intended target sequence; BPIV-3 M (membrane protein) (Horwood and Mahony, 2011; Kishimoto et al., 2017; Goto et al., 2020); BVDV 5′UTR (Goto et al., 2020) and 5′UTR (Kishimoto et al., 2017) (Supplementary Table S3).

Of the 32 primer sets analysed 75.0% returned a higher proportion of target-specific sequences than non-target (Supplementary Table S3). Five of the 32 primer sets analysed (15.6%) identified a greater proportion of non-target results than target-specific results. The majority of non-target hits had product lengths below 1000bp, therefore they could potentially be non-specifically amplified. Only three non-target hits: BPIV-3 Nucleoprotein gene (Thonur et al., 2012); BRSV nucleocapsid (Thonur et al., 2012) and N4 (Socha and Rola, 2011), had putative product lengths above 1000bp and therefore would be unlikely to result in non-specific amplification (Supplementary Table S3).

#### 3.1.2 Primer/Probe Sequences for Identification of Bacteria Associated With Respiratory Disease in Cattle

Of the 14 studies included in this review, 8 reported on the use of PCR for the detection of bacterial organisms associated with BRD (Szacawa et al., 2015; Nefedchenko et al., 2016; Kishimoto et al., 2017; Zhang et al., 2017; Loy et al., 2018; Thanthrige-Don et al., 2018; Andres-Lasheras et al., 2020; Goto et al., 2020). A total of 63 primer/probe sequences were extracted (probes n = 9, forward primers n = 27, reverse primers n = 27) and analysed using BLASTn (Madden, 2003) and Primer-Blast (Ye et al., 2012) (Table 6).

**TABLE 6:** Published PCR primer and probe sequences for detection of bacterial pathogens associated with bovine respiratory disease complex. F′ forward; P′ probe, R’ reverse sequences.

Pathogen	Target Gene	Sequence (5’—3′)	References
*Histophilus somni*	bamE (31 kDa)	F′ GCAATGATGTACCWGCCAAAG	(Loy et al., 2018; Goto et al., 2020)
		P′ TTG​CTT​ACG​TCC​AAA​CCG​TCG​TGT	
		R′ CCT​TCA​GCT​CAC​CAT​TAC​CAT​A	
	16S-rRNA	F′ AAG​GCC​TTC​GGG​TTG​TAA​AG	Kishimoto et al. (2017)
		P′ CGG​TGA​TGA​GGA​AGG​CGA​TTA​G
		R′ CCG​GTG​CTT​CTT​CTG​TGA​TTA​T
	16S-rDNA	F′ GTG​ATG​AGG​AAG​GCG​ATT​AGT	Thanthrige-Don et al. (2018)
		R′ TTCGGGCACCAAGTRTTCA
*Mannheimia haemolytica*	LktD	F′ CTG​CAA​CAA​AGC​CGA​TAT​CTT	(Loy et al., 2018; Goto et al., 2020)
		P′ ACA​CAT​CGT​CTT​CCG​GCA​CAA​TGA	
		R′ TAC​GAC​TGC​TGA​AAC​CTT​GAT	
	sodA	F′ ATT​AGT​GGG​TTG​TCC​TGG​TTA​G	Kishimoto et al. (2017)
		P′ CTG​AAC​CAA​CAC​GAG​TAG​TCG​CTG​C
		R′ GCG​TGA​TTT​CGG​TTC​AGT​TG
	LktA	F′ GTC​CCT​GTG​TTT​TCA​TTA​TAA​G	Thanthrige-Don et al. (2018)
		R′ CAC​TCG​ATA​ATT​ATT​CTA​AAT​TAG
	tbpB	F′ CTA​CTT​GCT​GCT​TGT​TCC​TC	Thanthrige-Don et al. (2018)
		R′ CCA​TGT​GCA​CCT​GTT​CTC​AAA
	nmaA	F′ AAG​CCG​TTT​CAA​CAT​TAG​CGT	Thanthrige-Don et al. (2018)
		R′ CAT​CGC​CAT​AAG​GGT​TGT​GA
	artJ-lktC	F′ TAT​AAG​GAT​TAC​CAC​TTT​AAC​GCA	Zhang et al. (2017)
		R′ ATA​ATC​AGA​AGA​GAA​AAA​GGA​GTG​T
	sodA	F′ GAC​TAC​TCG​TGT​TGG​TTC​AGG​CT	Nefedchenko et al. (2016)
		R′ CGG​ATA​GCC​TGA​AAC​GCC​T
*Mycoplasma bovis*	UvrC	F′ CCT​GTC​GGA​GTT​GCA​ATT​GT	Andres-Lasheras et al. (2020)
		R′ GCA​CTG​CGC​TCA​TTT​AAA​GC	
	OppD	F′ TCA​AGG​AAC​CCC​ACC​AGA​T	(Kishimoto et al., 2017; Loy et al., 2018; Goto et al., 2020)
		P′ TGG​CAA​ACT​TAC​CTA​TCG​GTG​ACC​CT
		R′ AGG​CAA​AGT​CAT​TTC​TAG​GTG​CAA
	16S-rDNA	F′ CCT​TTT​AGA​TTG​GGA​TAG​CGG​ATG	Thanthrige-Don et al. (2018)
		R′ CCG​TCA​AGG​TAG​CAT​CAT​TTC​CTA​T
	UvrC	F′ TTA​CGC​AAG​AGA​ATG​CTT​CA	Szacawa et al. (2015)
		R′ TAG​GAA​AGC​ACC​CTA​TTG​AT
*Pasteurella multocida*	Pm1231	F′ ATC​CCT​GCG​TTA​CAG​AGT​TTA​G	(Loy et al., 2018; Goto et al., 2020)
		P′ TTG​ATG​CCT​TCT​TTG​CGG​GTT​TCG	
		R′ GACGYGGGYAGTACCATAAA	
	kmt-1	F′ GGG​CTT​GTC​GGT​AGT​CTT​T	Kishimoto et al. (2017)
		P′ CGG​CGC​AAC​TGA​TTG​GAC​GTT​ATT
		R′ CGG​CAA​ATA​ACA​ATA​AGC​TGA​GTA
	Pm0762	F′ TTG​TGC​AGT​TCC​GCA​AAT​AA	Thanthrige-Don et al. (2018)
		R′ TTC​ACC​TGC​AAC​AGC​AAG​AC
	kmt-1	F′ TATCCGCTATTTACCCAG	Zhang et al. (2017)
		R′ TGTAAACGAACTCGCCAC
	kmt-1	F′ TAA​GAA​ACG​TAA​CTC​AAC​ATG​GAA​ATA	Nefedchenko et al. (2016)
		R′ GAG​TGG​GCT​TGT​CGG​TAG​TCT​T
	hyaD	F′ CGA​TAG​TCC​GTT​AGA​TAT​TGC​AAC	Nefedchenko et al. (2016)
		R′ CAT​AAT​GGA​TTT​GGC​GCC​AT
	dcbF	F′ ATC​GCA​TCC​AGA​ATA​GCA​AAC​TC	Nefedchenko et al. (2016)
		R′ TCC​GAT​GCT​TTG​GTT​GTG​C
	bcbD	F′ GCG​TGT​ATA​ACC​TAC​ATC​TTC​CCA	Nefedchenko et al. (2016)
		R′ CGT​CCA​TCA​ACA​CCT​TTA​CTG​C
	ecbJ	F′ TGGGCACATGCTCGCTTA	Nefedchenko et al. (2016)
		R′ CTG​CTT​GAT​TTT​GTC​TTT​CTC​CTA​A
	fcbD	F′ CGG​AGA​ACG​CAG​AAA​TCA​GAA	Nefedchenko et al. (2016)
		R′ CAA​CAA​CGA​CTT​CAA​ATG​GGT​AG
*Trueperella pyogenes*	plo-Pyolysin	F′ ATC​AAC​AAT​CCC​ACG​AAG​AG	Kishimoto et al. (2017)
		P′ TCG​ACG​GTT​GGA​TTC​AGC​GCA​ATA	
		R′ TTG​CAG​CAT​GGT​CAG​GAT​AC	
	plo	F′ CAG​TCA​AGG​GTG​AGT​CTA​TT	Zhang et al. (2017)
		R′ CTTGAACTCTGTGGAAA
*Ureaplasma diversum*	16S-rRNA	F′ CAT​TAA​ATG​ATG​TGC​CTG​GGT​AGT​AC	Kishimoto et al. (2017)
		P′ TTCGCAAGAATGAAAC	
		R′ CCCCGTCAATTCCGTTTG	

##### 3.1.2.1 BLASTn Analysis

**FIGURE 3:**
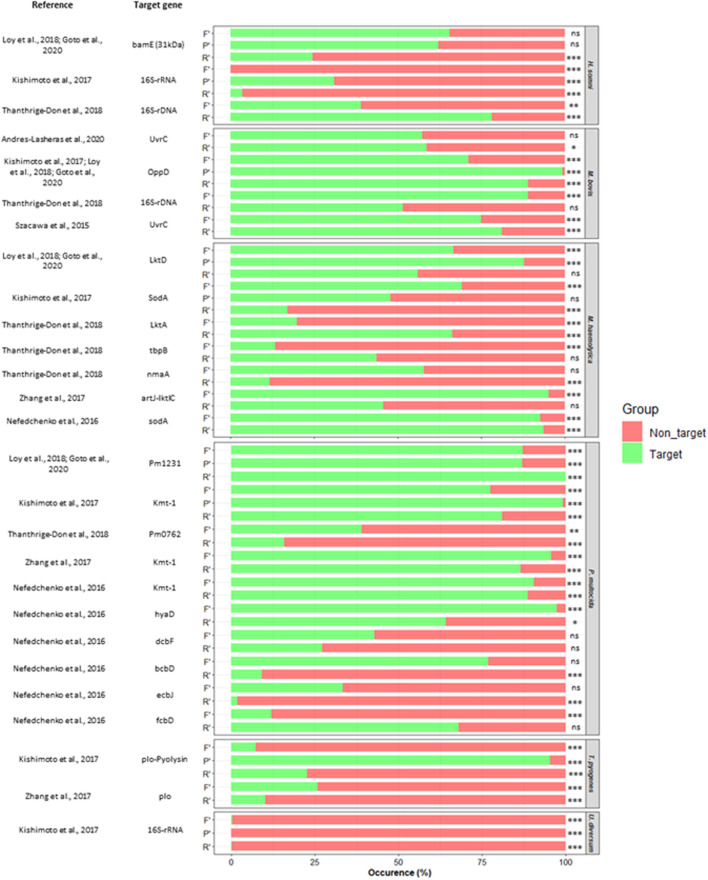
Occurrence of target and non-target sequence alignment for published primer (F′ forward, R′ reverse) and probe (P′) sequences targeting BRD associated bacterial pathogens *H. somni; Histophilus somni, P. multocida; Pasteurella multocida, T. pyogenes; Trueperella pyogenes, M. bovis; Mycoplasma bovis, M. haemolytica; Mannheimia haemolytica, U. diversum; Ureaplasma diversum.* Fisher’s exact test; *p* > 0.05 ns, *p* ≤ 0.05 *, *p* ≤ 0.01 **, *p* ≤ 0.001 ***.

The reverse primer targeting the Pm1231 gene of P*. multocida* (Loy et al., 2018; Goto et al., 2020) was the only sequence to be 100% target-specific. Of the 63 bacterial sequences extracted and analysed, 57.1% returned a higher number of target-specific results than non-targets. Of these, 77.8% had significantly more target-specific results than non-targets (Figure 3 and Supplementary Table S2). Twenty-seven of the 63 bacterial sequences analysed (42.9%) returned a higher number of non-target gene hits than target-specific results of which 21 had significantly more non-targets than target genes identified (Figure 3). The probe sequence for the 16srRNA gene of *U. diversum* was 100% specific for non-targets only and both forward and reverse primers for this target returned a higher number of non-target results than the target (Kishimoto et al., 2017) (Figure 3). The primers and probe targeting the 16s-rRNA gene of *H. somni* (Kishimoto et al., 2017) all had significantly more results for non-target genes than that target (Figure 3). While the primers targeting the tbpB gene of *M. haemolytica* (Thanthrige-Don et al., 2018), Pm0762 (Thanthrige-Don et al., 2018), dcbF (Nefedchenko et al., 2016) and ecbJ (Nefedchenko et al., 2016) genes of *P. multocida*, and *plo* gene of *T. pyogenes* (Zhang et al., 2017) all returned significantly more results for non-targets than the target (Figure 3).

##### 3.1.2.2 Primer-Blast Analysis of Bacterial Primer/Probe Sequences

Analysis of bacterial primer sets identified a number of primer pairs that showed 100% specificity for their intended target; specifically *H. somni* 16S rDNA (Thanthrige-Don et al., 2018); *M. haemolytica* tbpB and nmaA (Thanthrige-Don et al., 2018) and sodA (Nefedchenko et al., 2016); M. bovis oppD (Kishimoto et al., 2017; Loy et al., 2018; Goto et al., 2020); P. multocida Pm0762 (Thanthrige-Don et al., 2018), kmt-1 (Zhang et al., 2017), kmt-1, dcbF and bcbD (Nefedchenko et al., 2016); T. pyogenes plo-Pyolysin (Kishimoto et al., 2017) and plo (Zhang et al., 2017). Conversely, the primer pair targeting the *P. multocida* Pm1231 gene (Loy et al., 2018; Goto et al., 2020) reported no search results from Primer-Blast analysis (Supplementary Table S4).

Of the 27 primer sets analysed, 81.4% returned a higher number of target-specific results than non-target sequences whilst four primer pairs returned a significantly higher number of non-target hits than target-specific sequences (Supplementary Table S4). All non-target sequences identified that were either same genus and species as target but different strain, or a different genus but same target species (bovine) but a different bacterial species had product lengths below 1,000, potentially resulting in non-specifically amplification.Where primer pairs were identifying organisms of a completely different genus and species to the intended target, which was the case for reported sequences for *M. haemolytica* LktD (Loy et al., 2018; Goto et al., 2020), LktA (Thanthrige-Don et al., 2018) and artJ-lktC (Zhang et al., 2017); *M. bovis* uvrC (Andres-Lasheras et al., 2020) and UvrC (Szacawa et al., 2015); P. multocida kmt-1 (Kishimoto et al., 2017), hyaD (Nefedchenko et al., 2016), ecbJ (Nefedchenko et al., 2016) and fcbD (Nefedchenko et al., 2016) all non-target products had lengths above 1,000 and therefore would be unlikely to results in non-specific amplification (Supplementary Table S4) if the differential target was present in a mixed clinical sample.

Perhaps of most importance was the finding that some primer/probe sequences were found to be specific for non-target organisms that were also implicated as BRD pathogens. *Mannheimia haemolytica* was among the many non-target results detected during BLASTn analysis of sequences targeting the *P. multocida* Pm1231 gene (Loy et al., 2018; Goto et al., 2020). Similarly, *M. haemolytica* and *P. multocida* were among the non-target hits identified from BLASTn and Primer-Blast analysis of 16s-rRNA gene of *H. somni* (Kishimoto et al., 2017). This cross species reactivity for common co-habiting bacterial agents known to frequent the upper respiratory tract could lead to non-specific target application and therefore incorrect interpretation of quantitative results.

Together our findings suggest that reported primer sequences targeting BRD bacterial and viral pathogens may have questionable *in silico* specificity. This was found specifically, with the use of primer/probe sets targeting 16s rRNA sequences, which may result in cross-reactivity with other BRD pathogens in commonly used gene databases. It is possible that if this survey had been extended to all reports of primer/probe sequences used for the presence of, or genetic sequence analysis of bacterial and viral organisms associated with BRD in cattle, that results would show additional sequence disparity.

### 3.2 Reporting of Analytical and Diagnostic Sensitivity and Specificity

The application of PCR to disease diagnostics requires additional requirements to be considered above those required for research applications (Bustin et al., 2009). Definitions of analytical specificity and sensitivity and diagnostic specificity and sensitivity are shown in Table 1. To investigate whether specificity and sensitivity testing had been undertaken in relation to previously published results, articles were reviewed for evidence of both analytical and diagnostic sensitivity and specificity and the results tabulated (Table 3). Of the 14 articles analysed in this review, only one reported sensitivity and specificity for both analytical and diagnostic applications (Andres-Lasheras et al., 2020) (Table 3). All remaining articles reported analytical sensitivity and specificity but without confirming this in diagnostic samples (Table 3).

Three of the 14 studies analysed in this review reported on the use of quantitative PCR methods: qPCR (Andres-Lasheras et al., 2020), Multiplex qRT-PCR (Goto et al., 2020) and RT-qPCR (Xie et al., 2021) were indicated as techniques used but quantitative results were not reported in any report examined (Table 2).

### 3.3 Application of MIQE Guidelines

Quality and use of the MIQE guidelines were considered and reported methods were analysed against the twelve criteria outlined in Table 2. For practical reasons we focused on twelve criteria, which include the most critical MIQE parameters, however, this does not imply that the other MIQE parameters were absent. Of the ten studies reviewed, three were found to follow the MIQE guidelines fully. Of the reports investigated, only two studies (Kishimoto et al., 2017; Andres-Lasheras et al., 2020) referenced the Bustin et al. (2009) paper describing the MIQE guidelines. Kishimoto et al. (2017) referred to the MIQE paper in relation to the use of positive controls to evaluate PCR inhibition by nasal swab components, however, they failed to follow the guidelines in their methodology. In contrast, Andres-Lasheras et al. (2020) referred to the MIQE guidelines for developing the PCR assays used in their study and further supplied an in-depth outline of how their study followed the MIQE guidelines. Application and reporting on the application of the MIQE guidelines was therefore found to be inconsistent across the studies reviewed in this study.

## 4 Discussion

Veterinary diagnostics routinely adopt PCR based tests from the scientific literature to undertake research and diagnostics of animal diseases to support local livestock industries combat diseases that impact heavily on welfare and production economics. Whether research or clinical, reporting of the routine methodologies adopted to validate diagnostic standards is critical to ensure the reported tests have been rigorously validated and therefore that results are comparative and meaningful. Bovine Respiratory Disease (BRD) is a complex disease in cattle with numerous interacting etiological agents, environmental and physiological factors all contributing to risk, prevalence, and severity of disease globally (Richeson and Falkner, 2020). Like many veterinary diseases, PCR-based diagnostics are now routinely applied to confirm the presence of BRD disease-causing organisms and are often used as confirmatory diagnostic tests in combination with the veterinary clinical exam. The validity of these tests is therefore of paramount importance for the correct interpretation of results and comparative analysis between one report and another, but mostly, to ensure the test is fit for purpose.

To evaluate reported PCR techniques for the detection of pathogens associated with BRD, this review identified articles that reported the use of PCR assays, both quantitative and qualitative, used for identification of BRD-associated microorganisms, and analysed the *in silico* specificity of PCR primer/probe sequences as well as the application of the MIQE guidelines. Quantitative PCR is frequently suggested to provide quantitative data on the concentration of target sequences in a diagnostic or biological research sample. Despite the widespread use of the terminology, quantitation is often assumed, but not applied. The MIQE guidelines were developed in 2009 to tackle the lack of consensus on how best to perform and interpret quantitative real-time PCR (qPCR) experiments (Bustin et al., 2009). These guidelines also make recommendations on how to report quantitative PCR methodologies to improve the reader’s ability to evaluate results as either qualitative or quantitative (Bustin et al., 2009). Though the MIQE guidelines are made specifically for qPCR reporting, sections can be easily adopted for qualitative PCR, these sections of the MIQE guideline could include those used in Table 2 in this study. Of the 14 articles considered in this review, three articles fully applied the MIQE guidelines (Table 3). This may suggest that the guidelines have yet to be widely adopted in the field of bovine respiratory disease research, although a wider search of the literature related to all bovine diseases may have identified more reports that applied the MIQE guidelines. However, the fact that some articles applied the MIQE guidelines is a positive finding and suggests that uptake of these guidelines is now appearing in the animal health space as well as having widespread application in human diagnostics. Although the MIQE guidelines are specifically targeted at qPCR analyses, there is no reason that aspects of their experimental and reporting suggestions can’t be applied, where applicable, to qualitative PCR more broadly.

Sensitivity and specificity are core to the validation of any diagnostic or research methodology. Whilst all articles reviewed in this study reported analytical sensitivity and specificity testing, only one reported diagnostic testing. This indicated that the diagnostic performance of the PCR panels used may have not been confirmed prior to reporting of results. The majority of articles identified during screening were screened out due to failing to report sensitivity and specificity data for PCR-based diagnostics associated with BRD pathogens (n = 33, Figure 1). Failure to report analytical sensitivity and specificity could impede the interpretation of results. Again, it is possible that a more broadscale screen of the veterinary literature might identify more compliant articles, but the lack of reporting in this specific analysis suggests that adoption of reporting diagnostic, as well as analytical sensitivity and specificity in veterinary diagnostics, may be an area for future improvement.

When reported sequences were compared to the GenBank database using the BLASTn alignment (Madden, 2003) and Primer-Blast analysis (Ye et al., 2012), we found the *in silico* specificity of published primer/probe sequences to be highly variable. This is in comparison to a review of PCR assays designed to target human bacterial and viral sequences that had high *in silico* specificity, with almost no false positives (Lemmon and Gardner, 2008). There are several potential reasons for this difference between human and veterinary reports. Firstly, that there is a perception that the risk of false-positive or false-negative reporting in the veterinary field is low, and therefore rigorous *in silico* testing is not applied. Secondly, that unless the veterinary disease is a reportable disease there are minimal requirements to undertake significant scrutiny of reportable outcomes. And thirdly, that assumptions are made that the standard threshold reporting levels of *in silico* sensitivity provided by commonly used database search engines (generally first 100–200 sequences identified) are sufficiently rigorous to identify any undesirable targets. Our findings suggest that this may not be the case. Nagy et al. (2019) highlighted the need to periodically reassess assays *in silico* to not only determine specificity but ensure sensitivity (false negatives) have not changed as a result of the ongoing evolution of microbial pathogens and new additions to the sequences databases. Although *in silico* sensitivity was not tested in this review, the low *in silico* specificity of some of the reported assays suggests the prevalence of BRD associated pathogens may vary from the reported number if sequences with higher specificity had been used.

Several of the bacterial primer sequences tested in our *in silico*, using both BLASTn and Primer-Blast analysis, aligned not only with their target but with multiple other BRD associated microorganisms. This was specifically the case for reported sequences used to detect *H. somni*, *M. haemolytica* and *P. multocida*. This sequence cross-reactivity is likely due to similarities in their sequence identity due to a common phylogenetic origin as members of the Pasteurellaceae genus (Korczak et al., 2004). Our analysis showed a high level of variability with *in silico* sensitivity–from sequences that were 100% exclusive to the organism to be detected, to ones where a majority of non-target organism sequences aligned in the database (Figure 2 and Figure 3). Moreover, where 16S-rRNA sequences were used as a target gene, significant species cross-alignment was identified. This is likely due to the 16S-rRNA gene region containing both highly conserved and diverse regions between species which is present in all prokaryotes (Clarridge, 2004), suggesting that common sequences are likely between different species and genus of organisms. Using 16S-rRNA as the target can therefore make designing primers and probes that are impervious to non-specific binding challenging in PCR-based diagnostic assays and it is recommended that, where possible, other gene domains should be utilized for clinical or diagnostic reporting.

Many veterinary diagnostic laboratories worldwide offer in-house microbiological and PCR screening for BRD diagnostic purposes. More recently, commercially available PCR diagnostic kits for BRD associated organisms have become available including the VetMAX™ Ruminant Respiratory Screening Kit (Applied BioSystems™, United States), Pneumo 4™ (DNA Diagnostic A/S, Denmark) and RespoCheck (Central Veterinary Institute, Netherlands) and Kit Taqvet^®^ BRSV kits (L.S.I, France). Commercial kits do not report the processes of in-house testing, nor primer/probe sequences and, in some cases, target genes are not made available to the end-user. Where commercial tests were used in the reports considered in this study no analysis could be undertaken of their specificity and sensitivity for this reason. This also provides the third-party user with no opportunity to undertake independent *in silico* testing to confirm specificity therefore it is assumed that rigorous testing has been undertaken and standards met prior to commercialization of such kits. Whilst a number of studies have been published using commercially available diagnostic kits (Timsit et al., 2010; Cornelissen et al., 2017; Paller et al., 2017; Wisselink et al., 2017; Pansri et al., 2020), in house evaluations are rarely undertaken for these reasons and therefore these could not be evaluated in this study.

This study is the first to review, from a diagnostic methodological perspective, published reports utilizing PCR diagnostics for the identification and reporting of respiratory disease-causing organisms in cattle. We identified that some published PCR primer/probe assays used for the detection of bovine respiratory disease-causing organisms had questionable *in silico* specificity and, in some cases, the assays identified other bacteria implicated in the BRD disease complex. The authors recommend that broad utilization of the MIQE guidelines for both qualitative and quantitative PCR be adopted by animal scientists and veterinary researchers, and *in silico* testing reporting a high stringency of alignment to the intended target be an industry benchmark. Adopting these practices would decrease non-specific amplification and increase the reliability and reproducibility of reported assays when applying PCR diagnostic tests to disease-causing organisms in veterinary research and animal health studies.

## Data Availability

The original contributions presented in the study are included in the article/Supplementary Material, further inquiries can be directed to the corresponding author.
