# Supplementary material for: Meta-Analysis of qPCR for Bovine Respiratory Disease Based on MIQE Guidelines
Source: Front Mol Biosci. 2022 Jul 18;9:902401. doi: 10.3389/fmolb.2022.902401 (PMC9340069; doi:10.3389/fmolb.2022.902401)
Supplement: Supplementary file 1 [file Table1.DOCX]

Supplementary Material

# Supplementary Figures and Tables

## Supplementary Tables

Supplementary Table 1. Raw results from NCBI BLASTn search of BRD associated viral pathogens. Statistical significance was tested between proportion of target and non-target BLASTn results with coverage and identity above 95% (Fisher’s exact test). F’ forward primer, P’ probe, R’ reverse primer.

| **Pathogen** | **Target gene** | **Sequence (5’ – 3’)** | **Total number of search results with 95% coverage & identity or above** | **Number of target results with 95% coverage & identity or above** | **Number of non-target results with 95% coverage & identity or above** | **P- value** | **Reference** |
| --- | --- | --- | --- | --- | --- | --- | --- |
| Bovine Adenovirus 3 | Hexon | F’ ATTACCAGCGTCAACCTCTAC | 32 | 4 | 28 | <0.001 | (Kishimoto et al., 2017) |
|  |  | P’ TCCACTTTGGAAGCTATGCTCCGC | 12 | 5 | 7 | 0.732 |  |
|  |  | R’ CCGCCGAGAGATAGTCATTAAA | 17 | 4 | 13 | 0.081 |  |
| Bovine Adenovirus 7 | Hexon | F’ CRAGGGAATAYYTGTCTGAAAATC | 0 | 0 | 0 | NA | (Kishimoto et al., 2017) |
|  |  | P’ TTCATCWCTGCCACWCAAAGCTTTTTT | 53 | 5 | 48 | <0.001 |  |
|  |  | R’ AAGGATCTCTAAATTTYTCTCCAAGA | 51 | 5 | 46 | <0.001 |  |
| Bovine Coronavirus | N (nucleocapsid protein) | F’ GGACCCAAGTAGCGATGAG | 561 | 151 | 410 | <0.001 | (Kishimoto et al., 2017; Goto et al., 2020) |
|  |  | P’ ATTCCGACTAGGTTTCCGCCTGG | 579 | 155 | 424 | <0.001 |  |
|  |  | R’ GACCTTCCTGAGCCTTCAATA | 669 | 154 | 515 | <0.001 |  |
|  | N | F’ GCCGATCAGTCCGACCAATC | 323 | 162 | 161 | 1 | (Thanthrige-Don et al., 2018) |
|  |  | R’ AGAATGTCAGCCGGGGTAT | 220 | 132 | 88 | 0.016 |  |
| Bovine Herpes Virus 1 | gE (glycoprotein E) | F’ CAATAACAGCGTAGACCTGGTC | 76 | 68 | 8 | <0.001 | (Kishimoto et al., 2017; Goto et al., 2020) |
|  |  | P’ TGCGGCCTCCGGGCTTTACGTCT | 66 | 66 | 0 | <0.001 |  |
|  |  | R’ GCTGTAGTCCCAAGCTTCCAC | 117 | 61 | 56 | 0.735 |  |
|  | gC | F’ ATGTTAGCGCTCTGGAACC | 142 | 127 | 15 | <0.001 | (Horwood and Mahony, 2011) |
|  |  | P’ ACGGACGTGCGCGAAAAGA | 209 | 112 | 97 | 0.446 |  |
|  |  | R’ CTTTACGGTCGACGACTCC | 164 | 113 | 51 | <0.001 |  |
|  | Glycoprotein B gene | F’ TGAGGCCTATGTATGGGCAGTT | 82 | 58 | 24 | 0.003 | (Thanthrige-Don et al., 2018) |
|  |  | R’ GGACACAACAAACAATGCGG | 189 | 51 | 138 | <0.001 |  |
|  | Glycoprotein B gene | F’ TGTGGACCTAAACCTCACGGT | 82 | 55 | 27 | 0.014 | (Thonur et al., 2012) |
|  |  | P’ AGGACCGCGAGTTCTTGCCGC | 132 | 55 | 77 | 0.135 |  |
|  |  | R’ GTAGTCGAGCAGACCCGTGTC | 100 | 55 | 45 | 0.463 |  |
|  | gB | F’ GCGTCATTTACAAGGAGAACATC | 85 | 70 | 15 | <0.001 | (Fan et al., 2017) |
|  |  | R’ ATCTCGCCCATGCCCAC | 233 | 78 | 155 | <0.001 |  |
| Bovine Influenza D Virus | PB1 | F’ CAGCTGCGATGTCTGTCATAAG | 167 | 136 | 31 | <0.001 | (Kishimoto et al., 2017; Goto et al., 2020) |
|  |  | P’ AATGGACTTTCTCCTGGGACTGCT | 154 | 138 | 16 | <0.001 |  |
|  |  | R’ ACAAATTCGCAGGGCCATTA | 209 | 136 | 73 | <0.001 |  |
| Bovine Parainfluenza Virus 3 | M (membrane protein) | F’ TGTCTTCCACTAGATAGAGGGATAAAATT | 66 | 45 | 21 | 0.016 | (Horwood and Mahony, 2011; Kishimoto et al., 2017; Goto et al., 2020) |
|  |  | P’ ACAGCAATTGGATCAATAA | 157 | 21 | 136 | <0.001 |  |
|  |  | R’ GCAATGATAACAATGCCATGGA | 295 | 35 | 260 | <0.001 |  |
|  | M gene | F’ TGTCTTCCACTMGATAGAGGGATAAAATT | 47 | 43 | 4 | <0.001 | (Thanthrige-Don et al., 2018) |
|  |  | R’ CCTTTCTCATCTAAGATCTGGACMACC | 42 | 22 | 20 | 0.851 |  |
|  |  | F’ TGTCTTCCACTMGATAGAGGGATAAAATT | 47 | 43 | 4 | <0.001 |  |
|  |  | R’ CCTTTTTCATCTAGAATCTGAACTACTCC | 18 | 14 | 4 | 0.078 |  |
|  | Nucleoprotein gene | F’ GGTAGGAGCACCTCCACGATT | 64 | 36 | 28 | 0.078 | (Thonur et al., 2012) |
|  |  | P’ AAGATCTTGTTCACACATTC | 479 | 37 | 442 | <0.001 |  |
|  |  | R’ GCTCCAAGGCATGCTGGATA | 445 | 38 | 407 | <0.001 |  |
|  | Nucleoprotein gene | F’ TGATTGGATGTTCGGGAGTGA | 52 | 24 | 28 | 0.735 | (Thonur et al., 2012) |
|  |  | P’ TACAATCGAGGATCTTGTTCA | 86 | 28 | 58 | 0.008 |  |
|  |  | R’ AGAATCCTTTCCTCAATCCTGATATACT | 35 | 24 | 11 | 0.095 |  |
| Bovine Respiratory Syncytial Virus | N (nucleocapsid protein) | F’ GCAATGCTGCAGGACTAGGTATAAT | 53 | 14 | 39 | 0.006 | (Kishimoto et al., 2017; Goto et al., 2020) |
|  |  | P’ ACCAAGACTTGTATGATGCTGCCAAAGCA | 25 | 14 | 11 | 0.807 |  |
|  |  | R’ ACACTGTAATTGATGACCCCATTCT | 27 | 14 | 13 | 1 |  |
|  | N gene | F’ TATGCTATGTCCCGATTGG | 68 | 43 | 25 | 0.076 | (Liu et al., 2019) |
|  |  | R’ ACTGATTTGGCTAGTACACCC | 52 | 40 | 12 | 0.002 |  |
|  | G attachment glycoprotein | F’ ACACATCAATYCAAAGCACCACAC | 153 | 126 | 27 | <0.001 | (Thanthrige-Don et al., 2018) |
|  |  | R’ GCTRGTTCTGTGGTGGRTTGTTGTC | 72 | 70 | 2 | <0.001 |  |
|  | Nucleocapsid | F’ GGTCAAACTAAATGACACTTTCAACAAG | 41 | 14 | 27 | 0.124 | (Thonur et al., 2012) |
|  |  | P’ TAGTACAGGTGACAA+CA+T +TG | 261 | 14 | 247 | <0.001 |  |
|  |  | R’ AGCATACCACACAACTTATTGAGATG | 28 | 15 | 13 | <0.001 |  |
|  | Glycoprotein F | F’ AATCAACATGCAGTGCAGTTAG | 4837 | 8 | 4829 | <0.001 | (Socha and Rola, 2011) |
|  |  | R’ TTTGGTCATTCGTFATAGGCAT | 279 | 0 | 279 | <0.001 |  |
|  | Glycoprotein G | F’ CATCAATCCAAAGCACCACACTGTC | 123 | 109 | 14 | <0.001 | (Socha and Rola, 2011) |
|  |  | R’ GCTAGTTCTGTGGTGGATTGTTGTC | 29 | 21 | 8 | 0.066 |  |
|  | N4 | F’ GTTGCTGCTTTGGTTAT | 598 | 23 | 575 | <0.001 | (Socha and Rola, 2011) |
|  |  | R’ AGACTTGTATGATGCTGC | 29 | 13 | 16 | 0.82 |  |
|  | N protein | F’ GTCAGCTTAACATCAGAAGTTCAAG | 55 | 37 | 18 | 0.046 | (Socha and Rola, 2011) |
|  |  | R’ ACATAGCACTATCATACCACAATCA | 31 | 21 | 10 | 0.125 |  |
| Bovine Rhinitis A Virus | 3Dpol | F’ CACCTGAACTATGGACTTGG | 163 | 2 | 161 | <0.001 | (Kishimoto et al., 2017) |
|  |  | P’ GACGTGGACTGGCACCAGTTTGC | 9 | 9 | 0 | 0.012 |  |
|  |  | R’ CACGGCCTCAATCATCTG | 36 | 6 | 30 | <0.001 |  |
| Bovine Rhinitis B Virus | 3Dpol | F’ AACGCGATTGTGTCCTAGGG | 15 | 11 | 4 | 0.203 | (Kishimoto et al., 2017) |
|  |  | P’ CTGTCCTTTGCACGGCGTGG | 84 | 13 | 71 | <0.001 |  |
|  |  | R’ GCCACTGAGGTTAGCTTCTC | 184 | 10 | 174 | <0.001 |  |
|  | 3D gene | F’ CGTGGCACACTTCAGGAG | 18 | 8 | 10 | 0.777 | (Xie et al., 2021) |
|  |  | P’ TRGCRGGTCTCGCTTTYCACAGT | 0 | 0 | 0 | NA |  |
|  |  | R’ GTGTACCCAYCTCARACGAAG | 10 | 10 | 0 | 0.011 |  |
| Bovine Viral Diarrhea Virus | 5'UTR | F’ GRAGTCGTCARTGGTTCGAC | 0 | 0 | 0 | NA | (Goto et al., 2020) |
|  |  | P’ TGCYAYGTGGACGAGGGCATGC | 4 | 4 | 0 | 0.2081 |  |
|  |  | R’ TCAACTCCATGTGCCATGTAC | 1422 | 783 | 639 | 0.002 |  |
|  | 5'UTR | F’ TGGATGGCTTAAGCCCTGAGTA | 4915 | 4913 | 2 | <0.001 | (Horwood and Mahony, 2011) |
|  |  | P’ AGTCGTCAGTGGTTCGA | 4960 | 4958 | 2 | <0.001 |  |
|  |  | R’ CCTCGTCCACGTGGCATC | 4958 | 4942 | 16 | <0.001 |  |
|  | 5’UTR | F’ GGGNAGTCGTCARTGGTTCG | 4 | 4 | 0 | 0.2081 | (Kishimoto et al., 2017) |
|  |  | P’ CCAYGTGGACGAGGGCAYGC | 12 | 12 | 0 | 0.003 |  |
|  |  | R’ GTGCCATGTACAGCAGAGWTTTT | 1878 | 1266 | 612 | <0.001 |  |
|  | 5’UTR | F’ CATACCTTCAGTAGGACGAGC | 35 | 13 | 22 | 0.299 | (Thanthrige-Don et al., 2018) |
|  |  | R’ ATGTGCCATGTACAGCAGAG | 1908 | 1103 | 805 | <0.001 |  |
|  | 5’UTR | F’ CATGCCCRYAGTAGGACTAGC | 1 | 0 | 1 | 1 | (Thanthrige-Don et al., 2018) |
|  |  | R’ ATGTGCCATGTACAGCAGAG | 1908 | 1101 | 807 | <0.001 |  |
|  | 5’UTR | F’ GTGAGTTCGTTGGATGGC | 4974 | 4970 | 4 | <0.001 | (Fan et al., 2017) |
|  |  | R’ TATGTTTTGTATAAGAGTTCATTTG | 1448 | 1369 | 79 | <0.001 |  |

Supplementary Table 2. Raw results from NCBI BLASTn search of BRD associated bacterial pathogens. Statistical significance was tested between proportion of target and non-target BLASTn results with coverage and identity above 95% (Fisher’s exact test). F’ forward primer, P’ probe, R’ reverse primer.

| Pathogen | Target gene | Sequence (5’ – 3’) | Total number of search results with 95% coverage & identity or above | Number of target results with 95% coverage & identity or above | Number of non-target results with 95% coverage & identity or above | P- value | Reference |
| --- | --- | --- | --- | --- | --- | --- | --- |
| *Histophilus somni* | bamE (31kDa) | F’ GCAATGATGTACCWGCCAAAG | 32 | 21 | 11 | 0.192 | (Loy et al., 2018; Goto et al., 2020) |
|  |  | P’ TTGCTTACGTCCAAACCGTCGTGT | 53 | 33 | 20 | 0.177 |  |
|  |  | R’ CCTTCAGCTCACCATTACCATA | 134 | 33 | 101 | <0.001 |  |
|  | 16S-rRNA | F’ AAGGCCTTCGGGTTGTAAAG | 4997 | 1 | 4996 | <0.001 | (Kishimoto et al., 2017) |
|  |  | P’ CGGTGATGAGGAAGGCGATTAG | 258 | 80 | 178 | <0.001 |  |
|  |  | R’ CCGGTGCTTCTTCTGTGATTAT | 2978 | 105 | 2873 | <0.001 |  |
|  | 16S-rDNA | F’ GTGATGAGGAAGGCGATTAGT | 205 | 80 | 125 | 0.01 | (Thanthrige-Don et al., 2018) |
|  |  | R’ TTCGGGCACCAAGTRTTCA | 106 | 83 | 23 | <0.001 |  |
| *Mannheimia haemolytica* | LktD | F’ CTGCAACAAAGCCGATATCTT | 153 | 102 | 51 | <0.001 | (Loy et al., 2018; Goto et al., 2020) |
|  |  | P’ ACACATCGTCTTCCGGCACAATGA | 116 | 102 | 14 | <0.001 |  |
|  |  | R’ TACGACTGCTGAAACCTTGAT | 182 | 102 | 80 | 0.204 |  |
|  | sodA | F’ ATTAGTGGGTTGTCCTGGTTAG | 130 | 90 | 40 | <0.001 | (Kishimoto et al., 2017) |
|  |  | P’ CTGAACCAACACGAGTAGTCGCTGC | 188 | 90 | 98 | 0.656 |  |
|  |  | R’ GCGTGATTTCGGTTCAGTTG | 535 | 91 | 444 | <0.001 |  |
|  | LktA | F’ GTCCCTGTGTTTTCATTATAAG | 499 | 99 | 400 | <0.001 | (Thanthrige-Don et al., 2018) |
|  |  | R’ CACTCGATAATTATTCTAAATTAG | 149 | 99 | 50 | 0.001 |  |
|  | tbpB | F’ CTACTTGCTGCTTGTTCCTC | 396 | 53 | 343 | <0.001 | (Thanthrige-Don et al., 2018) |
|  |  | R’ CCATGTGCACCTGTTCTCAAA | 153 | 67 | 86 | 0.235 |  |
|  | nmaA | F’ AAGCCGTTTCAACATTAGCGT | 88 | 51 | 37 | 0.241 | (Thanthrige-Don et al., 2018) |
|  |  | R’ CATCGCCATAAGGGTTGTGA | 436 | 51 | 385 | <0.001 |  |
|  | artJ-lktC | F’ TATAAGGATTACCACTTTAACGCA | 104 | 99 | 5 | <0.001 | (Zhang et al., 2017) |
|  |  | R’ ATAATCAGAAGAGAAAAAGGAGTGT | 219 | 100 | 119 | 0.321 |  |
|  | sodA | F’ GACTACTCGTGTTGGTTCAGGCT | 97 | 90 | 7 | <0.001 | (Nefedchenko et al., 2016) |
|  |  | R’ CGGATAGCCTGAAACGCCT | 95 | 89 | 6 | <0.001 |  |
| *Mycoplasma bovis* | UvrC | F’ CCTGTCGGAGTTGCAATTGT | 258 | 148 | 110 | 0.057 | (Andres-Lasheras et al., 2020) |
|  |  | R’ GCACTGCGCTCATTTAAAGC | 262 | 154 | 108 | 0.023 |  |
|  | OppD | F’ TCAAGGAACCCCACCAGAT | 208 | 148 | 60 | <0.001 | (Kishimoto et al., 2017; Loy et al., 2018; Goto et al., 2020) |
|  |  | P’ TGGCAAACTTACCTATCGGTGACCCT | 150 | 149 | 1 | <0.001 |  |
|  |  | R’ AGGCAAAGTCATTTCTAGGTGCAA | 165 | 147 | 18 | <0.001 |  |
|  | 16S-rDNA | F’ CCTTTTAGATTGGGATAGCGGATG | 219 | 195 | 24 | <0.001 | (Thanthrige-Don et al., 2018) |
|  |  | R’ CCGTCAAGGTAGCATCATTTCCTAT | 392 | 202 | 190 | 0.665 |  |
|  | UvrC | F’ TTACGCAAGAGAATGCTTCA | 205 | 154 | 51 | <0.001 | (Szacawa et al., 2015) |
|  |  | R’ TAGGAAAGCACCCTATTGAT | 197 | 160 | 37 | <0.001 |  |
| *Pasteurella multocida* | Pm1231 | F’ ATCCCTGCGTTACAGAGTTTAG | 94 | 82 | 12 | <0.001 | (Loy et al., 2018; Goto et al., 2020) |
|  |  | P’ TTGATGCCTTCTTTGCGGGTTTCG | 100 | 87 | 13 | <0.001 |  |
|  |  | R’ GACGYGGGYAGTACCATAAA | 86 | 86 | 0 | <0.001 |  |
|  | kmt-1 | F’ GGGCTTGTCGGTAGTCTTT | 165 | 128 | 37 | <0.001 | (Kishimoto et al., 2017) |
|  |  | P’ CGGCGCAACTGATTGGACGTTATT | 129 | 128 | 1 | <0.001 |  |
|  |  | R’ CGGCAAATAACAATAAGCTGAGTA | 159 | 129 | 30 | <0.001 |  |
|  | Pm0762 | F’ TTGTGCAGTTCCGCAAATAA | 223 | 87 | 136 | 0.008 | (Thanthrige-Don et al., 2018) |
|  |  | R’ TTCACCTGCAACAGCAAGAC | 551 | 88 | 463 | <0.001 |  |
|  | kmt-1 | F’ TATCCGCTATTTACCCAG | 95 | 91 | 4 | <0.001 | (Zhang et al., 2017) |
|  |  | R’ TGTAAACGAACTCGCCAC | 119 | 103 | 16 | <0.001 |  |
|  | kmt-1 | F’ TAAGAAACGTAACTCAACATGGAAATA | 148 | 134 | 14 | <0.001 | (Nefedchenko et al., 2016) |
|  |  | R’ GAGTGGGCTTGTCGGTAGTCTT | 150 | 133 | 17 | <0.001 |  |
|  | hyaD | F’ CGATAGTCCGTTAGATATTGCAAC | 79 | 77 | 2 | <0.001 | (Nefedchenko et al., 2016) |
|  |  | R’ CATAATGGATTTGGCGCCAT | 120 | 77 | 43 | 0.013 |  |
|  | dcbF | F’ ATCGCATCCAGAATAGCAAACTC | 14 | 6 | 8 | 0.75 | (Nefedchenko et al., 2016) |
|  |  | R’ TCCGATGCTTTGGTTGTGC | 22 | 6 | 16 | 0.113 |  |
|  | bcbD | F’ GCGTGTATAACCTACATCTTCCCA | 13 | 10 | 3 | 0.169 | (Nefedchenko et al., 2016) |
|  |  | R’ CGTCCATCAACACCTTTACTGC | 108 | 10 | 98 | <0.001 |  |
|  | ecbJ | F’ TGGGCACATGCTCGCTTA | 3 | 1 | 2 | 1 | (Nefedchenko et al., 2016) |
|  |  | R’ CTGCTTGATTTTGTCTTTCTCCTAA | 55 | 1 | 54 | <0.001 |  |
|  | fcbD | F’ CGGAGAACGCAGAAATCAGAA | 108 | 13 | 95 | <0.001 | (Nefedchenko et al., 2016) |
|  |  | R’ CAACAACGACTTCAAATGGGTAG | 25 | 17 | 8 | 0.217 |  |
| *Trueperella pyogenes* | plo-Pyolysin | F’ ATCAACAATCCCACGAAGAG | 392 | 29 | 363 | <0.001 | (Kishimoto et al., 2017) |
|  |  | P’ TCGACGGTTGGATTCAGCGCAATA | 22 | 21 | 1 | <0.001 |  |
|  |  | R’ TTGCAGCATGGTCAGGATAC | 132 | 30 | 102 | <0.001 |  |
|  | plo | F’ CAGTCAAGGGTGAGTCTATT | 112 | 29 | 83 | <0.001 | (Zhang et al., 2017) |
|  |  | R’ CTTGAACTCTGTGGAAA | 217 | 22 | 195 | <0.001 |  |
| *Ureaplasma diversum* | 16S-rRNA | F’ CATTAAATGATGTGCCTGGGTAGTAC | 1101 | 5 | 1096 | <0.001 | (Kishimoto et al., 2017) |
|  |  | P’ TTCGCAAGAATGAAAC | 4995 | 0 | 4995 | <0.001 |  |
|  |  | R’ CCCCGTCAATTCCGTTTG | 1782 | 5 | 1777 | <0.001 |  |

**Supplementary Table 3.** PrimerBlast analysis for simultaneous assessment of forward and reverse primers targeting BRD associated viruses. Total number of PrimerBlast sequences analysed was determined by the PrimerBlast software. Target refers to the intended viral target and non-target is any other viral sequence. Same genus as target for all viral sequences refers to the genus *Bovine*; non-target species refers to an alternative viral species (i.e. Adenovirus). F’ forward primer, R’ reverse primer. BAdV, bovine adenovirus; BCoV, bovine coronavirus; BHV, bovine herpes virus; BIDV, bovine influenza D virus; BPIV, bovine parainfluenza virus; BRSV, bovine respiratory syncytial virus; BRAV, bovine rhinitis A virus; BRBV, bovine rhinitis B virus; BVDV, bovine viral diarrhea virus.

| **Target** | **Target gene** | **Sequence** | **Total Primer Blast sequences analysed** | **Total No. of hits** | **No. Target hits** | **Target product length** | **No. non-target hits** | **Break-down of distribution of non-target hits** | | | | | | | | **Reference** |
| --- | --- | --- | --- | --- | --- | --- | --- | --- | --- | --- | --- | --- | --- | --- | --- | --- |
|  |  |  |  |  |  |  |  | **Target genus & species, alternative strain** | **Product length** | **Different genus same viral species** | **Product length** | **Same genus different viral species** | **Product length** | **Different genus and viral species to target** | **Product length** |  |
| BAdV3 | Hexon | F’ ATTACCAGCGTCAACCTCTAC | 1304 | 4 | 4 | 121 | 0 | 0 | NA | 0 | NA | 0 | NA | 0 | NA | (Kishimoto et al., 2017) |
|  |  | R’ CCGCCGAGAGATAGTCATTAAA |  |  |  |  |  |  |  |  |  |  |  |  |  |  |
| BAdV7 | Hexon | F’ CRAGGGAATAYYTGTCTGAAAATC | 505 | 14 | 4 | 87 | 10 | 6 | 87 | 4 | 87 | 0 | NA | 0 | NA | (Kishimoto et al., 2017) |
|  |  | R’ AAGGATCTCTAAATTTYTCTCCAAGA |  |  |  |  |  |  |  |  |  |  |  |  |  |  |
| BCoV | N (nucleocapsid protein) | F’ GGACCCAAGTAGCGATGAG | 2511 | 629 | 157 | 90 | 472 | 0 | NA | 418 | 90 | 0 | NA | 54 | 90 | (Kishimoto et al., 2017; Goto et al., 2020) |
|  |  | R’ GACCTTCCTGAGCCTTCAATA |  |  |  |  |  |  |  |  |  |  |  |  |  |  |
|  | N | F’ GCCGATCAGTCCGACCAATC | 2316 | 237 | 159 | 407 | 78 | 0 | NA | 49 | 407 | 0 | NA | 29 | 407 | (Thanthrige-Don et al., 2018) |
|  |  | R’ AGAATGTCAGCCGGGGTAT |  |  |  |  |  |  |  |  |  |  |  |  |  |  |
| BHV-1 | gE (glycoprotein E) | F’ CAATAACAGCGTAGACCTGGTC | 3195 | 64 | 61 | 106 | 3 | 3 | 106 | 0 | NA | 0 | NA | 0 | NA | (Kishimoto et al., 2017; Goto et al., 2020) |
|  |  | R’ GCTGTAGTCCCAAGCTTCCAC |  |  |  |  |  |  |  |  |  |  |  |  |  |  |
|  | gC | F’ ATGTTAGCGCTCTGGAACC | 445 | 111 | 111 | 123 | 0 | 0 | NA | 0 | NA | 0 | NA | 0 | NA | (Horwood and Mahony, 2011) |
|  |  | R’ CTTTACGGTCGACGACTCC |  |  |  |  |  |  |  |  |  |  |  |  |  |  |
|  | Glycoprotein B gene | F’ TGAGGCCTATGTATGGGCAGTT | 2197 | 54 | 51 | 422-467 | 3 | 0 | NA | 0 | NA | 0 | NA | 3 | 265-513 | (Thanthrige-Don et al., 2018) |
|  |  | R’ GGACACAACAAACAATGCGG |  |  |  |  |  |  |  |  |  |  |  |  |  |  |
|  | Glycoprotein B gene | F’ TGTGGACCTAAACCTCACGGT | 1546 | 71 | 55 | 97 | 16 | 11 | 97 | 5 | 97 | 0 | NA | 0 | NA | (Thonur et al., 2012) |
|  |  | R’ GTAGTCGAGCAGACCCGTGTC |  |  |  |  |  |  |  |  |  |  |  |  |  |  |
|  | gB | F’ GCGTCATTTACAAGGAGAACATC | 4191 | 73 | 65 | 151 | 8 | 6 | 151 | 0 | NA | 0 | NA | 2 | 94-151 | (Fan et al., 2017) |
|  |  | R’ ATCTCGCCCATGCCCAC |  |  |  |  |  |  |  |  |  |  |  |  |  |  |
| BIDV | PB1 | F’ CAGCTGCGATGTCTGTCATAAG | 1680 | 180 | 180 | 83 | 0 | 0 | NA | 0 | NA | 0 | NA | 0 | NA | (Kishimoto et al., 2017; Goto et al., 2020) |
|  |  | R’ ACAAATTCGCAGGGCCATTA |  |  |  |  |  |  |  |  |  |  |  |  |  |  |
| BPIV-3 | M (membrane protein) | F’ TGTCTTCCACTAGATAGAGGGATAAAATT | 3312 | 0 | 0 | NA | 0 | 0 | NA | 0 | NA | 0 | NA | 0 | NA | (Horwood and Mahony, 2011; Kishimoto et al., 2017; Goto et al., 2020) |
|  |  | R’ ACAGCAATTGGATCAATAA |  |  |  |  |  |  |  |  |  |  |  |  |  |  |
|  | M gene | F’ TGTCTTCCACTMGATAGAGGGATAAAATT | 9223 | 524 | 37 | 203 | 487 | 0 | NA | 485 | 203 | 0 | NA | 2 | 203 | (Thanthrige-Don et al., 2018) |
|  |  | R’ CCTTTTTCATCTAGAATCTGAACTACTCC |  |  |  |  |  |  |  |  |  |  |  |  |  |  |
|  | M gene | F’ TGTCTTCCACTMGATAGAGGGATAAAATT | 1597 | 120 | 14 | 203 | 106 | 0 | NA | 106 | 203 | 0 | NA | 0 | NA | (Thanthrige-Don et al., 2018) |
|  |  | R’ CCTTTCTCATCTAAGATCTGGACMACC |  |  |  |  |  |  |  |  |  |  |  |  |  |  |
|  | Nucleoprotein gene | F’ GGTAGGAGCACCTCCACGATT | 1804 | 38 | 37 | 65 | 1 | 0 | NA | 0 | NA | 0 | NA | 1 | 2553 | (Thonur et al., 2012) |
|  |  | R’ GCTCCAAGGCATGCTGGATA |  |  |  |  |  |  |  |  |  |  |  |  |  |  |
|  | Nucleoprotein gene | F’ TGATTGGATGTTCGGGAGTGA | 3154 | 41 | 39 | 191 | 2 | 0 | NA | 2 | 191 | 0 | NA | 0 | NA | (Thonur et al., 2012) |
|  |  | P’ TACAATCGAGGATCTTGTTCA |  |  |  |  |  |  |  |  |  |  |  |  |  |  |
|  |  | R’ AGAATCCTTTCCTCAATCCTGATATACT |  |  |  |  |  |  |  |  |  |  |  |  |  |  |
| BRSV | N (nucleocapsid protein) | F’ GCAATGCTGCAGGACTAGGTATAAT | 5827 | 18 | 18 | 124 | 0 | 0 | NA | 0 | NA | 0 | NA | 0 | NA | (Kishimoto et al., 2017; Goto et al., 2020) |
|  |  | R’ ACACTGTAATTGATGACCCCATTCT |  |  |  |  |  |  |  |  |  |  |  |  |  |  |
|  | N gene | F’ TATGCTATGTCCCGATTGG | 634 | 46 | 46 | 596 | 0 | 0 | NA | 0 | NA | 0 | NA | 0 | NA | (Liu et al., 2019) |
|  |  | R’ ACTGATTTGGCTAGTACACCC |  |  |  |  |  |  |  |  |  |  |  |  |  |  |
|  | G attachment glycoprotein | F’ ACACATCAATYCAAAGCACCACAC | 607 | 95 | 95 | 374 | 0 | 0 | NA | 0 | NA | 0 | NA | 0 | NA | (Thanthrige-Don et al., 2018) |
|  |  | R’ GCTRGTTCTGTGGTGGRTTGTTGTC |  |  |  |  |  |  |  |  |  |  |  |  |  |  |
|  | Nucleocapsid | F’ GGTCAAACTAAATGACACTTTCAACAAG | 14147 | 25 | 23 | 138 | 2 | 0 | NA | 1 | 138 | 0 | NA | 1 | 3400 | (Thonur et al., 2012) |
|  |  | R’ AGCATACCACACAACTTATTGAGATG |  |  |  |  |  |  |  |  |  |  |  |  |  |  |
|  | Glycoprotein F | F’ AATCAACATGCAGTGCAGTTAG | 9670 | 15 | 14 | 711 | 1 | 0 | NA | 0 | NA | 0 | NA | 1 | 868 | (Socha and Rola, 2011) |
|  |  | R’ TTTGGTCATTCGTFATAGGCAT |  |  |  |  |  |  |  |  |  |  |  |  |  |  |
|  | Glycoprotein G | F’ CATCAATCCAAAGCACCACACTGTC | 2992 | 142 | 104 | 368-371 | 38 | 0 | NA | 0 | NA | 0 | NA | 38 | 265-309 | (Socha and Rola, 2011) |
|  |  | R’ GCTAGTTCTGTGGTGGATTGTTGTC |  |  |  |  |  |  |  |  |  |  |  |  |  |  |
|  | N4 | F’ GTTGCTGCTTTGGTTAT | 657 | 1 | 0 | NA | 1 | 0 | NA | 0 | NA | 0 | NA | 1 | 3884 | (Socha and Rola, 2011) |
|  |  | R’ AGACTTGTATGATGCTGC |  |  |  |  |  |  |  |  |  |  |  |  |  |  |
|  | N protein | F’ GTCAGCTTAACATCAGAAGTTCAAG | 2125 | 48 | 47 | 138 | 1 | 0 | NA | 1 | 138 | 0 | NA | 0 | NA | (Socha and Rola, 2011) |
|  |  | R’ ACATAGCACTATCATACCACAATCA |  |  |  |  |  |  |  |  |  |  |  |  |  |  |
| BRAV | 3Dpol | F’ CACCTGAACTATGGACTTGG | 566 | 2 | 2 | 171 | 0 | 0 | NA | 0 | NA | 0 | NA | 0 | NA | (Kishimoto et al., 2017) |
|  |  | R’ CACGGCCTCAATCATCTG |  |  |  |  |  |  |  |  |  |  |  |  |  |  |
| BRBV | 3Dpol | F’ AACGCGATTGTGTCCTAGGG | 452 | 12 | 12 | 112 | 0 | 0 | NA | 0 | NA | 0 | NA | 0 | NA | (Kishimoto et al., 2017) |
|  |  | R’ GCCACTGAGGTTAGCTTCTC |  |  |  |  |  |  |  |  |  |  |  |  |  |  |
|  | 3D gene | F’ CGTGGCACACTTCAGGAG | 242 | 9 | 9 | 144 | 0 | 0 | NA | 0 | NA | 0 | NA | 0 | NA | (Xie et al., 2021) |
|  |  | R’ GTGTACCCAYCTCARACGAAG |  |  |  |  |  |  |  |  |  |  |  |  |  |  |
| BVDV | 5’ UTR | F’ GRAGTCGTCARTGGTTCGAC | 2436 | 0 | 0 | NA | 0 | 0 | NA | 0 | NA | 0 | NA | 0 | NA | (Goto et al., 2020) |
|  |  | R’ TCAACTCCATGTGCCATGTAC |  |  |  |  |  |  |  |  |  |  |  |  |  |  |
|  | 5’ UTR | F’ TGGATGGCTTAAGCCCTGAGTA | 15902 | 1000 | 1000 | 85-87 | 0 | 0 | NA | 0 | NA | 0 | NA | 0 | NA | (Horwood and Mahony, 2011) |
|  |  | R’ CCTCGTCCACGTGGCATC |  |  |  |  |  |  |  |  |  |  |  |  |  |  |
|  | 5’ UTR | F’ GGGNAGTCGTCARTGGTTCG | 2636 | 0 | 0 | NA | 0 | 0 | NA | 0 | NA | 0 | NA | 0 | NA | (Kishimoto et al., 2017) |
|  |  | R’ GTGCCATGTACAGCAGAGWTTTT |  |  |  |  |  |  |  |  |  |  |  |  |  |  |
|  | 5’ UTR | F’ CATACCTTCAGTAGGACGAGC | 3316 | 20 | 19 | 282 | 1 | 0 | NA | 0 | NA | 1 | 274 | 0 | NA | (Thanthrige-Don et al., 2018) |
|  |  | R’ ATGTGCCATGTACAGCAGAG |  |  |  |  |  |  |  |  |  |  |  |  |  |  |
|  | 5’ UTR | F’ CATGCCCRYAGTAGGACTAGC | 3568 | 703 | 456 | 277-286 | 247 | 0 | NA | 0 | NA | 247 | 277-281 | 0 | NA | (Thanthrige-Don et al., 2018) |
|  |  | R’ ATGTGCCATGTACAGCAGAG |  |  |  |  |  |  |  |  |  |  |  |  |  |  |
|  | 5’UTR | F’ GTGAGTTCGTTGGATGGC | 42026 | 193 | 168 | 269-273 | 25 | 0 | NA | 0 | NA | 0 | NA | 25 | 427-2624 | (Fan et al., 2017) |
|  |  | R’ TATGTTTTGTATAAGAGTTCATTTG |  |  |  |  |  |  |  |  |  |  |  |  |  |  |

**Supplementary Table 4.** PrimerBlast analysis for simultaneous assessment of forward and reverse primers targeting BRD associated bacteria. Total number of PrimerBlast sequences analysed was determined by the PrimerBlast software. Target refers to the intended bacterial target. A non-target result is any other bacterial target irrespective of genus and species. Where non-target sequences are identified, these can be either the same genus as target but a different species, or an alternative genus and species. F’ forward primer, R’ reverse primer.

| **Target** | **Target gene** | **Sequence** | **Total Primer Blast sequences analysed** | **Total No. hits** | **No. target hits** | **Target hit product length** | **No. non-target hits** | **Break-down of distribution of non-target hits** | | | | | | **Reference** |
| --- | --- | --- | --- | --- | --- | --- | --- | --- | --- | --- | --- | --- | --- | --- |
|  |  |  |  |  |  |  |  | **Different genus same species** | **Product length** | **Same genus different species** | **Product length** | **Different genus and species to target** | **Product length** |  |
| *Histophilus somni* | bam E (31kDa antigen) | F’ GCAATGATGTACCWGCCAAAG | 1945 | 21 | 20 | 111 | 1 | 0 | NA | 0 | NA | 1 | 61 | (Loy et al., 2018; Goto et al., 2020) |
|  |  | R’ CCTTCAGCTCACCATTACCATA |  |  |  |  |  |  |  |  |  |  |  |  |
|  | 16S-rRNA | F’ AAGGCCTTCGGGTTGTAAAG | 127835 | 824 | 103 | 93 | 721 | 0 | NA | 0 | NA | 721 | 93 | (Kishimoto et al., 2017) |
|  |  | R’ CCGGTGCTTCTTCTGTGATTAT |  |  |  |  |  |  |  |  |  |  |  |  |
|  | 16S rDNA | F’ GTGATGAGGAAGGCGATTAGT | 2900 | 98 | 98 | 415-466 | 0 | 0 | NA | 0 | NA | 0 | NA | (Thanthrige-Don et al., 2018) |
|  |  | R’ TTCGGGCACCAAGTRTTCA |  |  |  |  |  |  |  |  |  |  |  |  |
| *Mannheimia haemolytica* | LktD | F’ CTGCAACAAAGCCGATATCTTT | 3166 | 130 | 114 | 95 | 16 | 0 | NA | 15 | 95 | 1 | 2576 | (Loy et al., 2018; Goto et al., 2020) |
|  |  | R’ TACGACTGCTGAAACCTTGAT |  |  |  |  |  |  |  |  |  |  |  |  |
|  | sodA | F’ ATTAGTGGGTTGTCCTGGTTAG | 2384 | 102 | 101 | 144 | 1 | 0 | NA | 1 | 144 | 0 | NA | (Kishimoto et al., 2017) |
|  |  | R’ GCGTGATTTCGGTTCAGTTG |  |  |  |  |  |  |  |  |  |  |  |  |
|  | LktA | F’ GTCCCTGTGTTTTCATTATAAG | 3752 | 113 | 110 | 381-395 | 3 | 0 | NA | 1 | 384 | 2 | 1056-1546 | (Thanthrige-Don et al., 2018) |
|  |  | R’ CACTCGATAATTATTCTAAATTAG |  |  |  |  |  |  |  |  |  |  |  |  |
|  | tbpB | F’ CTACTTGCTGCTTGTTCCTC | 2054 | 56 | 56 | 452-455 | 0 | 0 | NA | 0 | NA | 0 | NA | (Thanthrige-Don et al., 2018) |
|  |  | R’ CCATGTGCACCTGTTCTCAAA |  |  |  |  |  |  |  |  |  |  |  |  |
|  | nmaA | F’ AAGCCGTTTCAACATTAGCGT | 1472 | 53 | 53 | 396-399 | 0 | 0 | NA | 0 | NA | 0 | NA | (Thanthrige-Don et al., 2018) |
|  |  | R’ CATCGCCATAAGGGTTGTGA |  |  |  |  |  |  |  |  |  |  |  |  |
|  | artJ-lktC | F’ TATAAGGATTACCACTTTAACGCA | 7291 | 117 | 111 | 249-251 | 6 | 0 | NA | 0 | NA | 6 | 240-3703 | (Zhang et al., 2017) |
|  |  | R’ ATAATCAGAAGAGAAAAAGGAGTGT |  |  |  |  |  |  |  |  |  |  |  |  |
|  | sodA | F’ GACTACTCGTGTTGGTTCAGGCT | 1655 | 101 | 101 | 127 | 0 | 0 | NA | 0 | NA | 0 | NA | (Nefedchenko et al., 2016) |
|  |  | R’ CGGATAGCCTGAAACGCCT |  |  |  |  |  |  |  |  |  |  |  |  |
| *Mycoplasma bovis* | uvrC | F’ TTACGCAAGAGAATGCTTCA | 1071 | 162 | 161 | 1627 | 1 | 0 | NA | 0 | NA | 1 | 2977 | (Andres-Lasheras et al., 2020) |
|  |  | R’ TAGGAAAGCACCCTATTGAT |  |  |  |  |  |  |  |  |  |  |  |  |
|  | oppD | F’ TCAAGGAACCCCACCAGAT | 3913 | 161 | 161 | 71 | 0 | 0 | NA | 0 | NA | 0 | NA | (Kishimoto et al., 2017; Loy et al., 2018; Goto et al., 2020) |
|  |  | R’ AGGCAAAGTCATTTCTAGGTGCAA |  |  |  |  |  |  |  |  |  |  |  |  |
|  | 16S rDNA | F’ CCTTTTAGATTGGGATAGCGGATG | 3637 | 359 | 309 | 360 | 50 | 0 | NA | 50 | 359-361 | 0 | NA | (Thanthrige-Don et al., 2018) |
|  |  | R’ CCGTCAAGGTAGCATCATTTCCTAT |  |  |  |  |  |  |  |  |  |  |  |  |
|  | UvrC | F’ TTACGCAAGAGAATGCTTCA | 1071 | 162 | 161 | 1627-1630 | 1 | 0 | NA | 0 | NA | 1 | 2977 | (Szacawa et al., 2015) |
|  |  | R’ TAGGAAAGCACCCTATTGAT |  |  |  |  |  |  |  |  |  |  |  |  |
| *Pasteurella multocida* | Pm1231 | F’ ATCCCTGCGTTACAGAGTTTAG | 732 | 0 | 0 | NA | 0 | 0 | NA | 0 | NA | 0 | NA | (Loy et al., 2018; Goto et al., 2020) |
|  |  | R’ GACGYGGGYAGTACCATAAA |  |  |  |  |  |  |  |  |  |  |  |  |
|  | kmt-1 | F’ GGGCTTGTCGGTAGTCTTT | 5408 | 146 | 145 | 148 | 1 | 0 | NA | 0 | NA | 1 | 3331 | (Kishimoto et al., 2017) |
|  |  | R’ CGGCAAATAACAATAAGCTGAGTA |  |  |  |  |  |  |  |  |  |  |  |  |
|  | Pm0762 | F’ TTGTGCAGTTCCGCAAATAA | 2665 | 100 | 100 | 567-568 | 0 | 0 | NA | 0 | NA | 0 | NA | (Thanthrige-Don et al., 2018) |
|  |  | R’ TTCACCTGCAACAGCAAGAC |  |  |  |  |  |  |  |  |  |  |  |  |
|  | kmt-1 | F’ TATCCGCTATTTACCCAG | 348 | 107 | 107 | 456 | 0 | 0 | NA | 0 | NA | 0 | NA | (Zhang et al., 2017) |
|  |  | R’ TGTAAACGAACTCGCCAC |  |  |  |  |  |  |  |  |  |  |  |  |
|  | kmt-1 | F’ TAAGAAACGTAACTCAACATGGAAATA | 3350 | 145 | 145 | 211 | 0 | 0 | NA | 0 | NA | 0 | NA | (Nefedchenko et al., 2016) |
|  |  | R’ GAGTGGGCTTGTCGGTAGTCTT |  |  |  |  |  |  |  |  |  |  |  |  |
|  | hyaD | F’ CGATAGTCCGTTAGATATTGCAAC | 2328 | 77 | 76 | 564 | 1 | 0 | NA | 0 | NA | 1 | 1548 | (Nefedchenko et al., 2016) |
|  |  | R’ CATAATGGATTTGGCGCCAT |  |  |  |  |  |  |  |  |  |  |  |  |
|  | dcbF | F’ ATCGCATCCAGAATAGCAAACTC | 2801 | 6 | 6 | 356 | 0 | 0 | NA | 0 | NA | 0 | NA | (Nefedchenko et al., 2016) |
|  |  | R’ TCCGATGCTTTGGTTGTGC |  |  |  |  |  |  |  |  |  |  |  |  |
|  | bcbD | F’ GCGTGTATAACCTACATCTTCCCA | 648 | 11 | 11 | 168 | 0 | 0 | NA | 0 | NA | 0 | NA | (Nefedchenko et al., 2016) |
|  |  | R’ CGTCCATCAACACCTTTACTGC |  |  |  |  |  |  |  |  |  |  |  |  |
|  | ecbJ | F’ TGGGCACATGCTCGCTTA | 27320 | 10 | 1 | 358 | 9 | 0 | NA | 0 | NA | 9 | 532-3717 | (Nefedchenko et al., 2016) |
|  |  | R’ CTGCTTGATTTTGTCTTTCTCCTAA |  |  |  |  |  |  |  |  |  |  |  |  |
|  | fcbD | F’ CGGAGAACGCAGAAATCAGAA | 4202 | 16 | 15 | 258 | 1 | 0 | NA | 0 | NA | 1 | 3436 | (Nefedchenko et al., 2016) |
|  |  | R’ CAACAACGACTTCAAATGGGTAG |  |  |  |  |  |  |  |  |  |  |  |  |
| *Trueperella pyogenes* | plo-Pyolysin | F’ ATCAACAATCCCACGAAGAG | 1388 | 31 | 31 | 97 | 0 | 0 | NA | 0 | NA | 0 | NA | (Kishimoto et al., 2017) |
|  |  | R’ TTGCAGCATGGTCAGGATAC |  |  |  |  |  |  |  |  |  |  |  |  |
|  | plo | F’ CAGTCAAGGGTGAGTCTATT | 453 | 22 | 22 | 773 | 0 | 0 | NA | 0 | NA | 0 | NA | (Zhang et al., 2017) |
|  |  | R’ CTTGAACTCTGTGGAAA |  |  |  |  |  |  |  |  |  |  |  |  |
| *Ureaplasma diversum* | 16S-rRNA | F’ CATTAAATGATGTGCCTGGGTAGTAC | 17126 | 997 | 5 | 62 | 992 | 0 | NA | 115 | 62 | 877 | 62 | (Kishimoto et al., 2017) |
|  |  | R’ CCCCGTCAATTCCGTTTG |  |  |  |  |  |  |  |  |  |  |  |  |

**References**

Andres-Lasheras, S., Zaheer, R., Ha, R., Lee, C., Jelinski, M., and McAllister, T.A. (2020). A direct qPCR screening approach to improve the efficiency of Mycoplasma bovis isolation in the frame of a broad surveillance study. *Journal of Microbiological Methods* 169. doi: 10.1016/j.mimet.2019.105805.

Fan, Q., Xie, Z., Xie, Z., Deng, X., Xie, L., Huang, L., et al. (2017). Development of a GeXP-multiplex PCR assay for the simultaneous detection and differentiation of six cattle viruses. *PLoS One* 12(2). doi: http://dx.doi.org/10.1371/journal.pone.0171287.

Goto, Y., Yaegashi, G., Fukunari, K., and Suzuki, T. (2020). Design of a multiplex quantitative reverse transcription‐PCR system to simultaneously detect 16 pathogens associated with bovine respiratory and enteric diseases. *Journal of Applied Microbiology* 129(4)**,** 832-847. doi: 10.1111/jam.14685.

Horwood, P.F., and Mahony, T.J. (2011). Multiplex real-time RT-PCR detection of three viruses associated with the bovine respiratory disease complex. *Journal of Virological Methods* 171(2)**,** 360-363. doi: 10.1016/j.jviromet.2010.11.020.

Kishimoto, M., Tsuchiaka, S., Rahpaya, S.S., Hasebe, A., Otsu, K., Sugimura, S., et al. (2017). Development of a one-run real-time PCR detection system for pathogens associated with bovine respiratory disease complex. *Journal of Veterinary Medical Science* 79(3)**,** 517-523.

Liu, Z., Li, J., Liu, Z., Li, J., Li, Z., Wang, C., et al. (2019). Development of a nanoparticle-assisted PCR assay for detection of bovine respiratory syncytial virus. *BMC Veterinary Research* 15(1)**,** N.PAG-N.PAG. doi: 10.1186/s12917-019-1858-0.

Loy, J.D., Bulut, E., Clawson, M.L., Leger, L., Wang, B., and Workman, A.M. (2018). Development of a multiplex real-time PCR assay using two thermocycling platforms for detection of major bacterial pathogens associated with bovine respiratory disease complex from clinical samples. *Journal of veterinary diagnostic investigation* 30(6)**,** 837-847. doi: http://dx.doi.org/10.1177/1040638718800170.

Nefedchenko, A.V., Shikov, A.N., Glotov, A.G., Glotova, T.I., Ternovoy, V.A., Agafonov, A.P., et al. (2016). Development of a method for identification and genotyping of Pasteurella multocida and Mannheimia haemolytica bacteria using polymerase chain reaction and phylogenetic analysis of bacterial cultures isolated from cattle. *Molecular Genetics Microbiology and Virology* 31(2)**,** 75-81. doi: 10.3103/s0891416816020063.

Socha, W., and Rola, J. (2011). Comparison of four RT-PCR assays for detection of bovine respiratory syncytial virus. *Polish Journal of Veterinary Sciences* 14(3)**,** 449-451.

Szacawa, E., Niemczuk, K., Dudek, K., Bednarek, D., Rosales, R., and Ayling, R. (2015). Mycoplasma bovis infections and co-infections with other Mycoplasma spp. with different clinical manifestations in affected cattle herds in eastern region of Poland. *Journal of Veterinary Research* 59(3)**,** 331-338. doi: http://dx.doi.org/10.1515/bvip-2015-0049.

Thanthrige-Don, N., Lung, O., Furukawa-Stoffer, T., Buchanan, C., Joseph, T., Godson, D.L., et al. (2018). A novel multiplex PCR-electronic microarray assay for rapid and simultaneous detection of bovine respiratory and enteric pathogens. *Journal of virological methods* 261**,** 51-62. doi: http://dx.doi.org/10.1016/j.jviromet.2018.08.010.

Thonur, L., Maley, M., Gilray, J., Crook, T., Laming, E., Turnbull, D., et al. (2012). One-step multiplex real time RT-PCR for the detection of bovine respiratory syncytial virus, bovine herpesvirus 1 and bovine parainfluenza virus 3. *BMC Veterinary Research* 8(1)**,** 37-45. doi: 10.1186/1746-6148-8-37.

Xie, Y.L., Lv, D.A.H., Wen, X.H., Zhai, Q., Luo, M.L., Wei, W.K., et al. (2021). Development of a Real-Time Quantitative RT-PCR Assay for Detection of Bovine Rhinitis B Virus. *Frontiers in Veterinary Science* 8**,** 6. doi: 10.3389/fvets.2021.680707.

Zhang, W., Liu, X., Liu, M., Ma, B., Xu, L., and Wang, J. (2017). Development of a multiplex PCR for simultaneous detection of Pasteurella multocida, Mannheimia haemolytica and Trueperella pyogenes. *Acta veterinaria Hungarica* 65(3)**,** 327-339. doi: http://dx.doi.org/10.1556/004.2017.032.
